# De novo assembly provides new insights into the evolution of *Elaeagnus angustifolia* L.

**DOI:** 10.1186/s13007-022-00915-w

**Published:** 2022-06-18

**Authors:** Yunfei Mao, Xueli Cui, Haiyan Wang, Xin Qin, Yangbo Liu, Yijun Yin, Xiafei Su, Juan Tang, Fengling Wang, Fengwang Ma, Naibin Duan, Donglin Zhang, Yanli Hu, Wenli Wang, Shaochong Wei, Xiaoliu Chen, Zhiquan Mao, Xuesen Chen, Xiang Shen

**Affiliations:** 1 College of Horticultural Science and Engineering/State Key Laboratory of Crop Biology, Shandong Agricultural University, Tai’an, China; 2 Biomarker Technologies Corporation, Beijing, China; 3 College of Horticulture, Northwest Agriculture and Forestry University, Yangling, China; 4 Germplasm Resource Center of Shandong Province, Shandong Academy of Agricultural Sciences, Jinan, China; 5 Department of Horticulture, University of Georgia, Athens, USA

**Keywords:** *Elaeagnus angustifolia* L., Genome, Evolutionary response mechanism, Desertification-affected land

## Abstract

**Background:**

*Elaeagnus angustifolia* L. is a deciduous tree in the family *Elaeagnaceae.* It is widely used to study abiotic stress tolerance in plants and to improve desertification-affected land because of its ability to withstand diverse types of environmental stress, such as drought, salt, cold, and wind. However, no studies have examined the mechanisms underlying the resistance of *E. angustifolia* to environmental stress and its adaptive evolution.

**Methods:**

Here, we used PacBio, Hi-C, resequencing, and RNA-seq to construct the genome and transcriptome of *E. angustifolia* and explore its adaptive evolution.

**Results:**

The reconstructed genome of *E. angustifolia* was 526.80 Mb, with a contig N50 of 12.60 Mb and estimated divergence time of 84.24 Mya. Gene family expansion and resequencing analyses showed that the evolution of *E. angustifolia* was closely related to environmental conditions. After exposure to salt stress, GO pathway analysis showed that new genes identified from the transcriptome were related to ATP-binding, metal ion binding, and nucleic acid binding.

**Conclusion:**

The genome sequence of *E. angustifolia* could be used for comparative genomic analyses of *Elaeagnaceae* family members and could help elucidate the mechanisms underlying the response of *E. angustifolia* to drought, salt, cold, and wind stress. Generally, these results provide new insights that could be used to improve desertification-affected land.

**Supplementary Information:**

The online version contains supplementary material available at 10.1186/s13007-022-00915-w.

## Background

The world’s population is increasing rapidly and is projected to reach 9.6 billion by 2050 [1]. Hence, global food production will need to increase 38 and 57% by 2025 and 2050, respectively, to maintain the current level of food supply [2]. However, the world’s irrigated land is decreasing by 1–2% annually [3], and soil degradation due to salinization is one of the major causes of this reduction in irrigated land [4]. More than 1125 million hectares of land worldwide are salt-affected, of which approximately 76 million hectares are affected by human-induced salinization and sodification [4]. Salinity stress is a major abiotic stress affecting plant growth and crop productivity [5]. Soil salinization is a major cause of land degradation, and it can make land unsuitable for crop cultivation [6]. In recent years, biological measures have been shown to be some of the most effective approaches for ameliorating salt-affected soil [7].

**Fig. 1:**
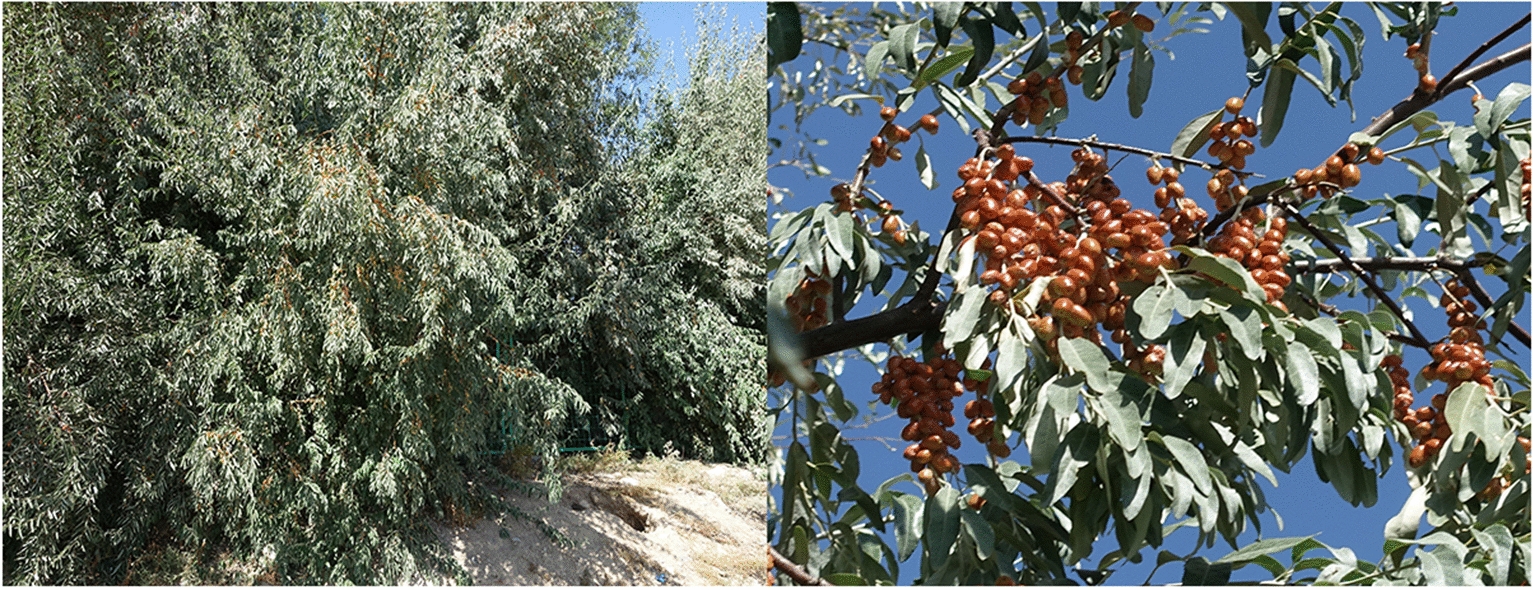
*E. angustifolia* in the south campus of Shandong Agricultural University, Tai’an, Shandong, China

*E. angustifolia* L., also known as Russian olive, is a deciduous tree belonging to the family *Elaeagnaceae* (Fig. 1). It is native to central and western Africa and is distributed in the United States, Canada, the Mediterranean coast, southern Russia, Iran, and India. It is widely distributed in China and occurs in several provinces including Xinjiang, Gansu, Ningxia, and Shandong [8]. The fruit of *E. angustifolia* is rich in sugars, flavonoids, and other substances that can regulate the circulation of blood and immune function in humans; the branches, leaves, and flowers have anti-aging properties and can be used to treat burns, bronchitis, dyspepsia, and neurasthenia [9]. *E. angustifolia* is tolerant of drought, salt, cold, and wind stress. It is prolific and highly adaptable, as it can grow in a variety of climates and soils [10]. *E. angustifolia* can grow and reproduce normally in soil with a salt content of 0.8–1.2% [11]. As a nitrogen-fixing, actinorrhizal plant, *E. angustifolia* is likely an early successional, pioneer species that can colonize nitrogen-poor soils such as sandy, eroded mineral soils and wetlands [12]. Consequently, *E. angustifolia* has often been used for the reforestation of arid and salinized zones [13].

Although the development of genome sequencing has aided the domestication and improvement of many species [14], relatively few studies have used genomic tools to study *E. angustifolia*. Ghodhbane-Gtari et al. [15] reporteds the 11.3-Mbp draft genome sequence of *Frankia* sp. strain BMG5.11 in *E. angustifolia*, which had a G + C content of 69.9% and 9926 candidate protein-encoding genes. Lin et al. [16] conducted a genome-wide transcriptome analysis and found that high salt concentration inhibited the growth and photosynthesis of *E. angustifolia*, which was caused by the down-regulation of genes encoding key enzymes involved in photosynthesis and genes related to important structures in the photosystems and light-harvesting complexes. However, no studies to date have examined the reference genome of *E. angustifolia*. Furthermore, little is known about the mechanisms of adaptive evolution and transcription of *E. angustifolia* under salt stress.

Here, we used PacBio, Hi-C assisted assembly, resequencing, and other technologies to explore the adaptive evolution of *E. angustifolia*. We used the transcriptome to explore the mechanism by which *E. angustifolia* responds to salt stress. The results of this study provide new insights that could be used to aid the planting of *E. angustifolia*, increase food income, and promote recovery from global land desertification.

## Materials and methods

### Sample collection and DNA extraction

Samples from *E. angustifolia* trees (obtained from Xinjiang Province, NCBI Taxonomic ID, 36777) were collected from the south campus of Shandong Agricultural University, Tai’an, Shandong, China (36.17101°N, 117.16074°E, 134.0 masl) (Fig. 1) for genomic DNA sequencing, Hi-C assisted assembly, and genome evolution and transcriptome analyses. Twelve wild *E. angustifolia* samples were collected for genome resequencing in Gansu, China (Table 1). After collection, samples were immediately immersed in liquid nitrogen and stored until DNA extraction. DNA was extracted using the Cetyltrimethyl Ammonium Bromide (CTAB) method. The quality of the extracted genomic DNA was assessed using 1% agarose gel electrophoresis [17], and the concentration was quantified using a Qubit fluorimeter (Invitrogen Co., Carlsbad, CA, USA).

**Table 1:** Wild *E. angustifolia* samples used in this study for whole-genome resequencing

Population	Code ID	Sample ID	Group	Country	Province	City	Coordinates	Altitude (m)
*E. angustifolia*	R01	1583	G1	China	Gansu	Gulang	37°29ʹ25ʺN; 102°54ʹ37ʺE	2026.8
	R02	1619	G1	China	Gansu	Jinchang	38°21ʹ25ʺN; 102°8ʹ24ʺE	1704.6
	R03	1684	G1	China	Gansu	Linze	38°58ʹ1ʺN; 99°54ʹ52ʺE	1900.3
	R04	1697	G2	China	Gansu	Jia Yuguan	39°31ʹ28ʺN; 98°51ʹ8ʺE	1531.1
	R05	1726	G2	China	Gansu	Jiuquan	39°33ʹ37ʺN; 98°51ʹ37ʺE	1457.4
	R06	1758	G2	China	Gansu	Jiuquan	39°41ʹ40ʺN; 98°35ʹ55ʺE	1433.6
	R07	1784	G2	China	Gansu	Jiuquan	39°41ʹ44ʺN; 98°35ʹ57ʺE	1473.0
	R08	1811	G2	China	Gansu	Zhangye	39°41ʹ42ʺN; 99°35ʹ33ʺE	1450.1
	R09	1857	G3	China	Gansu	Zhangye	39°47ʹ14ʺN; 98°14ʹ36ʺE	1695.0
	R10	2063	G3	China	Gansu	Dunhuang	40°7ʹ55ʺN; 94°54ʹ48ʺE	1756.2
	R11	2106	G3	China	Gansu	Guazhou	40°22ʹ3ʺN; 99°54ʹ52ʺE	1133.0
	R12	2216	G3	China	Gansu	Lanzhou	36°29ʹ21ʺN; 103°33ʹ22ʺE	1902.8

The numbers (R01, R02, R03, R04, R05, R06, R07, R08, R09, R10, R11, R12) represent 12 wild *E. angustifolia* samples. G1, G2, and G3 were short for Group 1, 2, and 3, respectively

### Genomic sequencing

The library was constructed after evaluating the quantity and quality of DNA samples. Two 270-bp paired-end libraries were prepared according to the Illumina protocol and sequenced on the Illumina HiSeq 2000 platform (Biomarker Technologies Co., Ltd., Beijing, China). Whole-genome sequencing was performed using the PacBio Sequel sequencer (Biomarker Technologies Co., Ltd., Beijing, China). Twelve 20-kb single-molecule real-time sequencing (SMRT) bell libraries were constructed using a PacBio DNA Template Prep Kit 1.0 (Vazyme Biotech Co., Ltd., Nanjing, Jiangsu, China). The templates were size-selected, and the BluePippin devices (Sage Science, Inc., Annoron, Beijing, China) were used to enrich long snippets (> 10 kb). The PacBio Sequel platform processed a total of 39 single cells per molecule in real time (SMRT).

### Genome assembly and genome annotation

A kmer map of k = 19 was constructed using the two 270-bp libraries and used to evaluate genome size, repeat sequence ratio, and heterozygosity. The formula used was G = (N k-mer—Nerror_k-mer)/D, where G is genome size, N k-mer is the number of k-mers, Nerror_k-mer is the number of k-mers with the depth of 1, and D is the k-mer depth.

All low-quality sequences shorter than 500 bp were removed through the PacBio sequencing platform (Biomarker Technologies Co., Ltd., Beijing, China). The results of Canu’s [18] assembly and the wtdbg (https://github.com/ruanjue/wtdbg) assembly were merged using Quickmerge [19]. The homologous contigs of the merged genomes were determined by comparison of the two genome assemblies, and the short-read data were used to error correct the merged genome with Pilon [20].

State programs [21] were used to construct a database of repetitive sequences for the *E. angustifolia* genome. EVM v1.1.1 [20] software was then used to create a consensus repeat library. Genscan [22] and other programs were used for ab initio gene model prediction. GeMoMa v1.3.1 [23] was used for homology-based gene prediction. Hisat v2.0.4 [24] and Stringtie v1.2.3 [24] were used for assembly based on reference transcripts. TransDecoder v2.0 [25] and GeneMarkS-T v5.1 [26] were used for gene prediction. PASA v2.0.2 [27] was used for the prediction of unigene sequences based on transcriptome data without a reference assembly. Finally, EVM v1.1.1 was used to integrate the prediction results obtained by the above three methods. Based on the Rfam [28] database and miRBase [29] database and Infenal 1.1 [30] for rRNA and microRNA prediction, tRNAscan-SE v1.3.1 [31] was used to identify tRNAs. BLAST v2.2.31 [22] with an E-value cutoff of 1E-5 was used to align the predicted gene sequences with functional databases such as Gene Ontology (GO) [32] and Kyoto Encyclopedia of Genes and Genomes (KEGG) [33].

The Core Eukaryotic Gene Mapping Approach (CEGMA) v2.5 [34] database contains 458 conserved core genes in eukaryotes. CEGMA was used to evaluate the completeness of the final genome assembly. The embryophyta_odb9 database in BUSCO v2 [35] contains 1440 conserved core genes in terrestrial plants. We used BUSCO software to evaluate the completeness of the *E. angustifolia* genome assembly.

### Hi-C analysis and assembly

Formaldehyde was used to fix the fresh *E. angustifolia* tissue samples. The DNA was digested with the restriction enzyme *Hind*III; after adding biotin-labeled bases, the repaired DNA was circularized, de-crosslinked, and purified, followed by fragmentation into 300–700-bp fragments. Streptavidin magnetic beads were used to capture the DNA fragments showing interaction relationships for library construction. Qubit2.0 (Life Technologies, CA, USA) and Agilent 2100 (Agilent Technologies) were used to detect the concentration and insert size of the library, and qPCR was used to quantify the effective concentration of the library.

The Illumina high-throughput sequencing platform was used to sequence the Hi-C library to produce a large number of high-quality reads. BWA (version: 0.7.10-r789; alignment mode: aln; other parameters default) [36] was used to compare the sequencing paired-end data with the sequences of the assembled genome. The contigs of the draft genome were split into simulated 500-kb contigs, and LACHESIS software [37] was used to cluster these contigs into groups.

### Genome evolution and gene family expansion

We used the single-copy protein sequences of seven other species, *Solanum lycopersicum*, *Arabidopsis thaliana* (Linn) Heynh, *Populus trichocarpa* Torr & Gray, *Glycine max* (Linn) Merr, *Oryza sativa* Linn, *Amborella trichopoda* Baill, and *Ziziphus jujuba* M, to build phylogenetic trees using PHYML software [38]. The divergence times between *E. angustifolia* and the seven other sequenced species were estimated using MrBayes60 and the Mcmctree [39] programs implemented in the Phylogenetic Analysis by Maximum Likelihood (PAML) 61 software package. Calibration times were obtained from the TimeTree database (http://www.timetree.org/) with ‘(*A. trichopoda*, *O*. *sativa*) ‘< 199 > 173’, (*A. thaliana*, *P. trichocarpa*) ‘< 117 > 98’, (*Z. jujuba*, *E. angustifolia*) ‘< 117 > 79’, and (*G. max*, *E. angustifolia*) ‘< 113 > 89’. We then calculated the four-fold synonymous third-codon transversion (4DTv) values.

OrthoMCL [40] software was used to cluster the protein sequences of *E. angustifolia* and seven other sequenced species. CAFE [41] was used to analyze gene family contraction and expansion. Functional enrichment analysis was used to identify the function of genes over-represented in our genome assembly. GO enrichment analysis and KEGG annotation were performed using R scripts [42]. CodeML [43] was used in PAML to detect the rapidly evolving genes in *E. angustifolia* with a single copy shared by all species.

### Genome resequencing

The code IDs of 12 wild *E. angustifolia* samples were R01–R12 (Table 1). The raw reads obtained by sequencing were filtered, low-quality reads with adapters were removed, and clean reads were obtained for subsequent information analysis. We used single nucleotide polymorphisms (SNPs) and small insertions and deletions (small Indels) to detect differences between our 12 wild *E. angustifolia* and the reference genome. The demographic history of 12 wild *E. angustifolia* was inferred using a hidden Markov model approach as implemented in the Pairwise Sequentially Markovian Coalescent (PSMC) model based on the SNP distribution [44]. We scaled results to real time, assuming 2 years per generation and a neutral mutation rate of 7.31 × 10^–9^ (CI 95% Poisson distribution: 5.20 × 10^–9^ ~ 8.00 × 10^–9^) per generation [45]. Genes with SNPs and Indels were analyzed by functional annotation. Polymorphic genes in the 12 wild *E. angustifolia* samples were compared with BLAST, GO, KEGG, and other functional databases to evaluate their function. MEGA X [46] software was used to construct phylogenetic trees with the neighbor-joining method. Admixture [47] software was used to analyze the group structure of the samples. The number of subgroups (K value) was preset to 1–10 for clustering; the clustering results were cross-validated, and the optimal number of clusters was determined according to the valley value of the cross-validation error rate. We artificially divided the 12 wild *E. angustifolia* species into three groups (G1–G3) based on factors such as latitude and longitude of the sampling sites (Table 1), and we designated the outgroup *Z. jujuba* as G0. PCA was performed on 12 wild *E. angustifolia* species and *Z. jujuba*.

### Salinity experiment

A greenhouse experiment was conducted in 2018 at State Key Laboratory of Crop Biology, Shandong Agriculture University, Tai’an, China. In March 2018, we mixed 200 seeds (collected from the *E. angustifolia* field in the breeding experimental base of Shandong Agricultural University) and wet sand into woven bags and placed them in an outdoor leeward shelter for lamination. We prepared 45 clay pots with an outer diameter of 29 cm, an inner diameter of 25 cm, and a depth of 18 cm. Each pot contained 15 kg of sterilized soil [48]. Three seeds were planted in each pot after one-third of the seeds had germinated in April 2018.

**Fig. 2:**
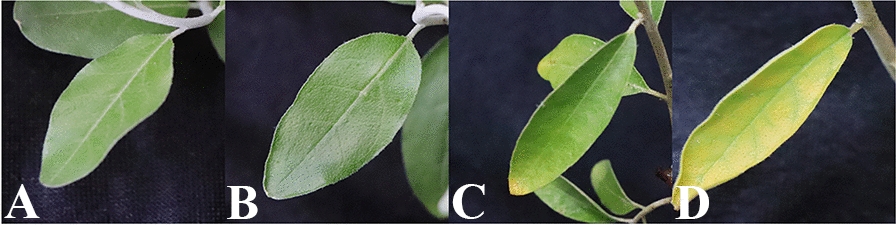
Treatment of *E. angustifolia* under salt stress for 10 days. The letters in pictures represent the electrical conductivity of different NaCl analytical reagent solutions. **A** 12 dS m^−1^; **B** 16 dS m^−1^; **C** 20 dS m^−1^; **D** 24 dS m^−1^

Irrigation of all pots was carried out for 2 months using normal urban water (salinity of 0.6 dS m^−1^) at field capacity. When seedlings were well established two months after planting, four concentrations (electrical conductivity of 12 dS m^−1^, 16 dS m^−1^, 20 dS m^−1^, and 24 dS m^−1^) of NaCl analytical reagent (Keephway Technologies Corporation, Beijing, China) solution were used for the salt stress treatment; 5 pots of *E. angustifolia* were randomly selected for each treatment following the methods of Zeng et al. [49]. The other 25 pots were watered using normal urban water. Observations of the leaves of the four test groups were made every day to determine the level of salt stress (Fig. 2). Salinity stress continued for 10 days when the salinity toxicity in the leaves was over 20 dS m^−1^. Salinity treatment was initiated with 25 pots by NaCl analytical reagent (Keephway Technologies Corporation, Beijing, China) solution with 20 dS m^−1^. We randomly divided 25 pots into 5 treatments (Group 1, 2, 3, 4, 5). The leaves of *E. angustifolia* in Group 1 were collected immediately (control group including T01&T02&T03); the leaves in Group 2 were collected 1 h after treatment (salinity group including T04&T05&T06); the leaves in Group 3 were collected 6 h after treatment (salinity group including T07&T08&T09); the leaves in Group 4 were collected 12 h after treatment (salinity group including T10&T11&T12); and the leaves in Group 5 were collected 24 h after treatment (salinity group including T13&T14&T15). All fresh leaf samples were immediately dissected and submerged in RNA later^®^ solution and stored in a − 80 °C freezer for RNA extraction. During the experiment, the maximum temperature was 31.0 ± 5.0 °C, and the minimum temperature was 19.5 ± 4.5 °C.

### RNA extraction, library construction, and RNA sequencing

The total RNA of each sample was extracted from the leaves of *E. angustifolia* using the RNA plant Plus Reagent (Vazyme Biotech Co., Ltd., Nanjing, Jiangsu, China). The RNA integrity and concentration were assessed using an Agilent 2100 Bioanalyzer (Agilent Technologies, Inc., Santa Clara, CA, USA). mRNA was isolated by the NEBNext Poly (A) mRNA Magnetic Isolation Module (NEB, E7490). The cDNA library was constructed using the NEBNext Ultra RNA Library Prep Kit for Illumina (NEB, E7530) and NEBNext Multiplex Oligos for Illumina (NEB, E7500) per the manufacturer’s instructions. Briefly, the enriched mRNA was fragmented into approximately 200-nt RNA inserts, which were used to synthesize first-strand cDNA and second-strand cDNA. End-repair/dA-tail and adaptor ligation were performed on the double-stranded cDNA. The suitable fragments were isolated by Agencourt AMPure XP beads (Beckman Coulter, Inc.) and enriched by PCR amplification. Finally, the constructed cDNA libraries of *E. angustifolia* were sequenced on a flow cell using the Illumina HiSeq™ sequencing platform.

### Transcriptome analysis using reference genome-based reads mapping

Low-quality reads, such as those containing adaptors, with unknown nucleotides > 5%, or Q20 < 20% (percentage of sequences with sequencing error rates < 1%), were removed using perl scripts. The clean reads that were filtered from the raw reads were mapped to the *E. angustifolia* genome (OGSv3.2) using Tophat2 [50]. The aligned records from the aligners in BAM/SAM format were further examined to remove potential duplicate molecules. Gene expression levels were estimated using FPKM values (fragments per kilobase of exon per million fragments mapped) by Cufflinks software [51].

### Identification of differentially expressed genes

DESeq2 [52] and Q-value were used to identify differentially expressed genes between Group 1 and Groups 2, 3, 4, and 5. Differences in gene abundance between these samples were calculated based on the ratio of the FPKM values. The false discovery rate (FDR) control method was used to identify the P-value threshold to evaluate the significance of differences. Here, only genes with an absolute value of log2 ratio ≥ 2 and FDR significance score < 0.01 were used in subsequent analyses.

### Sequence annotation

Differentially expressed genes were compared against various protein databases by BLASTX, including the National Center for Biotechnology Information (NCBI) non-redundant protein (Nr) database and Swiss-Prot database with a cut-off E-value of 10^–5^. Furthermore, genes were searched against the NCBI non-redundant nucleotide sequence (Nt) database using BLASTn with a cut-off E-value of 10^–5^. Genes were retrieved based on the best BLAST hit (highest score) along with their protein functional annotation.

The Nr BLAST results were imported into the Blast2 GO program [53]. GO annotations for the genes were obtained by Blast2GO. In this analysis, all of the annotated genes were mapped to GO terms in the database, and the number of genes associated with each term was counted. A perl script was then used to plot the GO functional classification for the unigenes to visualize the distribution of gene functions. The obtained annotations were enriched and refined using the TopGo (R package). The gene sequences were also aligned to the Clusters of Orthologous Group (COG) database to predict and classify functions [54]. KEGG pathways were assigned to the assembled sequences by perl scripts.

## Results and discussion

### Genome assembly of *E. angustifolia*

One band was visible following 1% agarose gel electrophoresis, and the concentration of DNA extracted was approximately 474.3 mg L^−1^. A total of 5,125,675 subreads were obtained by filtering low-quality data, and a total of 44.27 Gb raw PacBio sequel reads were obtained, with an average length of 8.64 kb (Additional file 1: Table S1). The subread N50 was 12,635 bp, and the average length was 8636 bp (Additional file 1: Table S2). After merging and correcting the assemblies, the final estimated genome size was 526.80 Mb, and the contig N50 was 12.60 Mb (Additional file 1: Table S3).

**Fig. 3:**
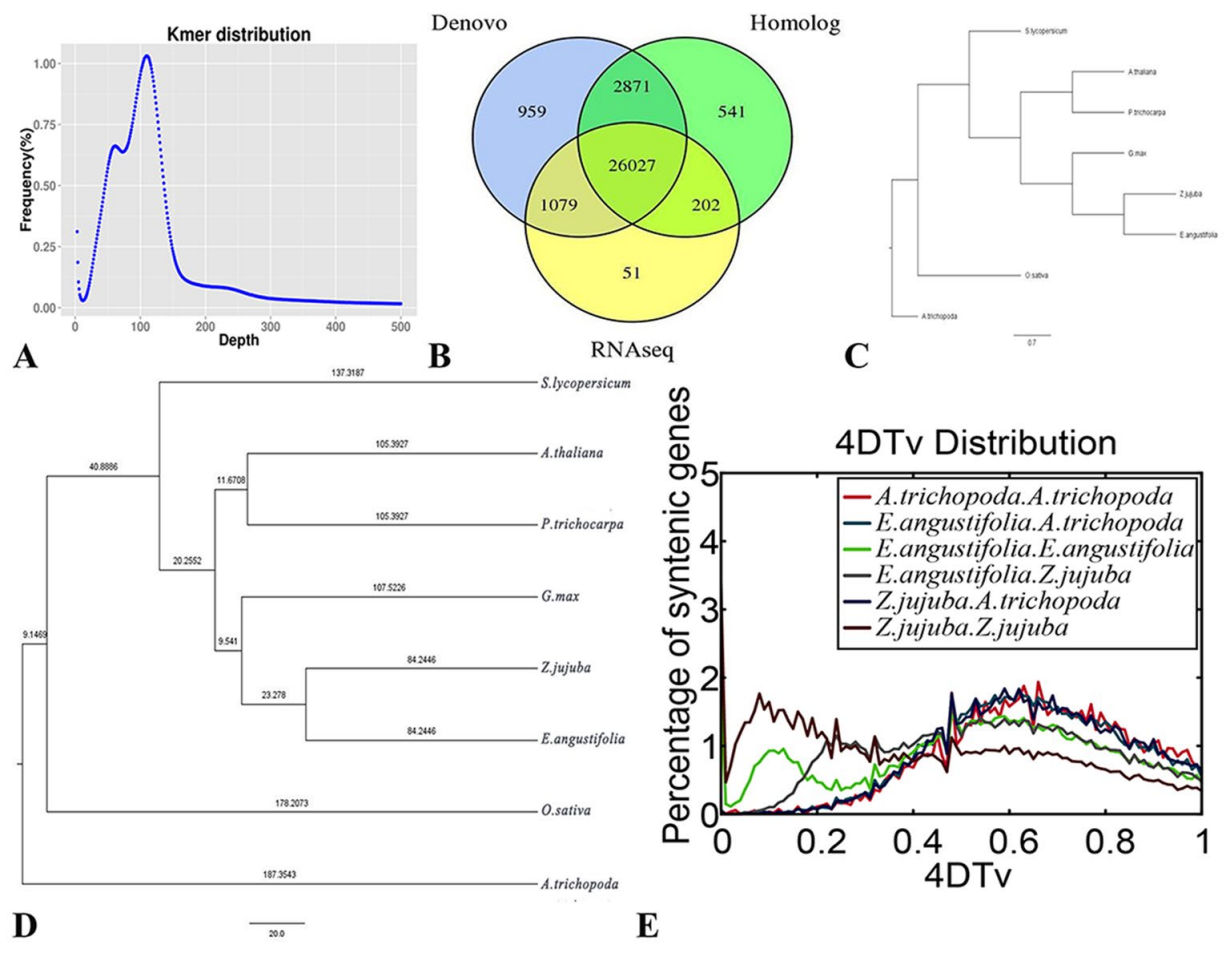
Genome assembly and genome evolution. **A** K-mer distribution of genome sequencing reads of *E. angustifolia*. K-mers (K = 19) were extracted from the paired-end library with an insert size of 270 bp. The total 23-mer count is 51,064,317,165. The peak 19-mer depth is 111, and the genome size was calculated as 51,064,317,165/111 = 456.24 Mb. **B** The integrated gene is derived from the distribution map of three prediction methods. **C** Phylogenetic tree of eight species **D** Estimation of the divergence time between *E. angustifolia* and other species. The number in each branch is the divergence time from the present (in million years ago). **E** Distribution of 4DTV values among *Z. jujuba*, *E. angustifolia*, and *A. trichopoda* genomes

The total number of k-mers was 153,631,375,991, with a k-mers peak at a depth of 111 (Fig. 3A), and the final genome had a heterozygosity estimate of 1.47%. We constructed a specific repeat sequence database for the prediction of repeat sequences for specific species, and the prediction yielded approximately 263.44 Mb of repeats without overlap, accounting for 50.01% of the total length of repeat sequences (Additional file 1: Table S4). A total of 31,730 (Additional file 1: Table S5) protein-coding genes and 127 miRNAs were annotated by integrating different methods (Additional file 1: Table S6). There were 30,771 genes available for transcriptome sequencing, accounting for 96.98% of all genes (Fig. 3B). A total of 96.89% of the genes could be annotated to NR and other databases (Additional file 1: Table S7). Conserved CEGMA analyses indicated that 97.38% of the core protein-coding genes were recovered in our assembled genome (Additional file 1: Table S8). BUSCO revealed 1290 complete gene models out of 1,440 (89.58%); 23 fragmented and 184 complete genes were found in duplicate (Additional file 1: Table S9).

### Hi-C assisted assembly

We obtained 39.56 Gb clean reads, with a sequencing coverage of 75 × and Q30 ratio of > 95.46% (Additional file 1: Table S10). The ratio of reads mapped to the assembled genome was 90.68%, and the ratio of unique mapped read pairs was 61.13%, indicating that the Hi-C data were suitable for subsequent analysis (Additional file 1: Table S11). A total of 80.79 M pairs of reads from the genome were obtained in this experimental library. Among them, 72.98 M pairs were valid Hi-C data, accounting for 90.32% of the data from the genome, and the ratio of invalid interaction pairs was 9.68% (Additional file 1: Table S12).

*E. angustifolia* has 14 chromosomes. The genome was also visualized at the chromosome level. After Hi-C assembly, a total of 510.71 Mb of genomic sequences were mapped to the chromosomes, accounting for 96.94% of the total length of the sequences, and the corresponding number of sequences was 269, accounting for 45.83% of the total number of sequences. Among the sequences located on the chromosomes, the sequence length that could determine the order and direction of chromosomes was 473.91 Mb, accounting for 92.8% of the total length of the sequences located on the chromosomes, and the number of corresponding sequences was 104, accounting for 38.66% of the total number of sequences located on the chromosomes (Table 2).

**Table 2:** The genomic sequence of *E. angustifolia* mapped to the chromosomes

Group	Sequence count	Sequence length **(**bp**)**
Lachesis group 0	28	27,993,989
Lachesis group 1	41	99,096,873
Lachesis group 2	23	57,474,495
Lachesis group 3	12	48,501,719
Lachesis group 4	28	30,089,891
Lachesis group 5	7	26,462,114
Lachesis group 6	28	18,656,685
Lachesis group 7	10	24,577,875
Lachesis group 8	8	26,266,316
Lachesis group 9	8	24,034,829
Lachesis group 10	9	27,579,915
Lachesis group 11	19	32,396,042
Lachesis group 12	26	37,035,969
Lachesis group 13	22	30,539,364
Total sequences clustered (ratio %)	269 (45.83)	510,706,076 (96.94)
Total sequences ordered and oriented (ratio %)	104 (38.66)	473,912,606 (92.8)

The statistics were all based on sequence lengths of 1 Kb or more

### Genome evolution of *E. angustifolia*

Comparative genome analysis harnesses the power of sequence comparisons within and between species to infer evolutionary history and provide information on the function of specific DNA sequences [55]. The deep-level evolutionary history of *E. angustifolia* has not yet been clarified. In our study, a phylogenetic tree (Fig. 3C, D) revealed that the origins of *A. trichopoda*, *O. sativa*, and *S. lycopersicum* were dated to 187.35, 178.21, and 137.32 million years ago (Mya), respectively. The origins of *A. thaliana* in the family *Brassicaceae* and *P. trichocarpa* in the family *Salicaceae* were dated to 137.32 Mya; *G. max* (107.52 Mya) in the family *Leguminosae* was in the same branch as *Z. jujuba* in the family *Rhamnaceae* and *E. angustifolia* in the family *Elaeagnaceae.* The origins of *Z. jujuba* and *E. angustifolia* were both dated to 84.24 Mya. These findings are similar to those of Harkess et al. [56].

To further analyze the evolutionary divergence of *E. angustifolia* and other species, 4DTv rates were calculated (Fig. 3E). The highest 4DTV value of *A. trichopoda* was 0.68, indicating that no recent genome-wide duplication has occurred. The 4DTV value peak of *Z. jujuba* was 0.08, and that of *E. angustifolia* was 0.12, indicating that both *Z. jujuba* and *E. angustifolia* have undergone recent genome-wide replication. Both 4DTv values of the autopolyploid of *Z. jujuba* and *E. angustifolia* peaked at 0.48, indicating that the time of the polyploid splitting event of *Z. jujuba* and *E. angustifolia* was similar to the time before the divergence of *Rosaceae* and *Elaeagnaceae*. The orthologs between *E. angustifolia* and *Z. jujuba* and between *E. angustifolia* and *A. trichopoda* indicated that the 4DTv values peaked at 0.25 and 0.48, respectively, suggesting that the divergence between *E. angustifolia* and *A. trichopoda* occurred earlier.

### Expanded gene families related to stress adaptation in *E. angustifolia*

**Fig. 4:**
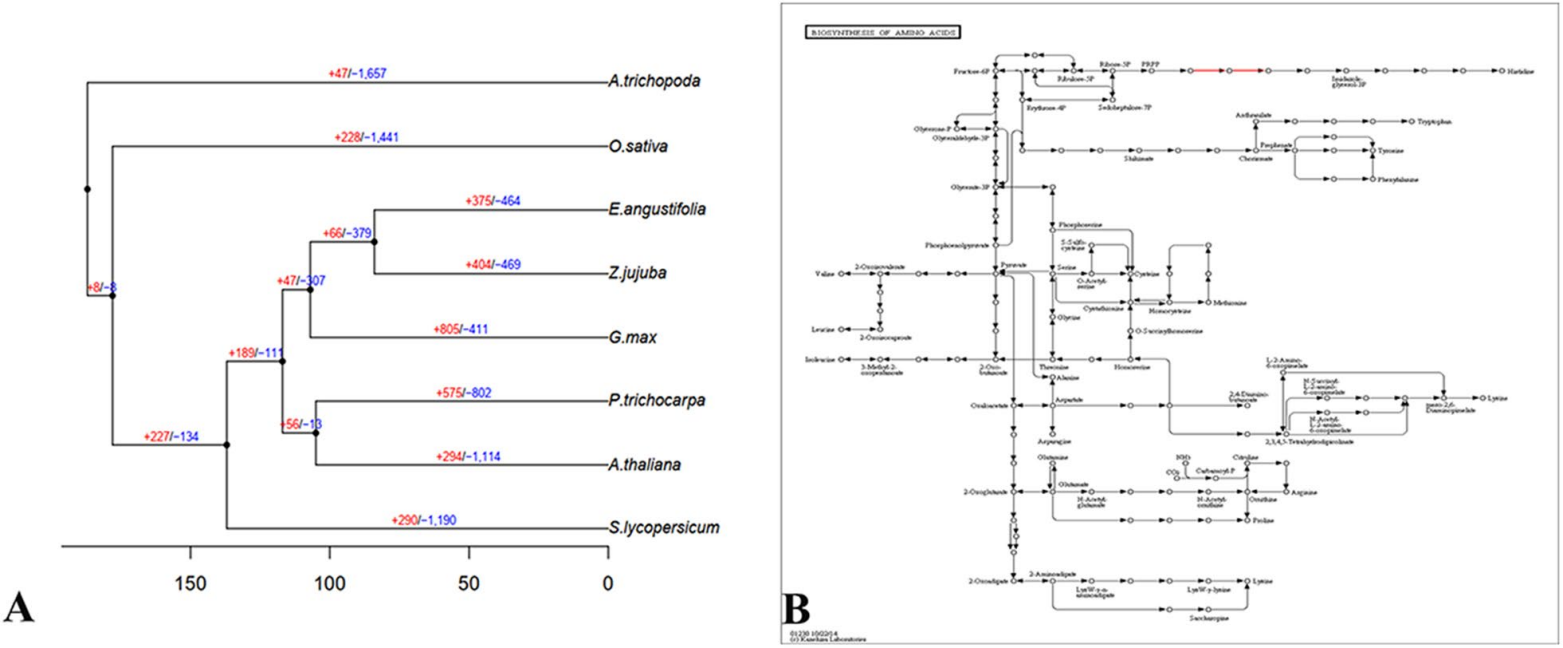
Expanded gene families. **A** Expansion (red numbers) and contracted (blue numbers) gene families in different plants; “ + ” represents the number of gene families that have expanded on the node; and “−” represents the number of gene families that have contracted on the node. Black dots indicate a common ancestor. **B** Biosynthesis of the amino acid regulation pathway in the KEGG analysis of *E. angustifolia*

The identification of expanded gene families provides valuable insights into the biological innovation and adaptive evolution of *E. angustifolia*. A total of 27,553 of 31,730 genes of *E. angustifolia* could be classified into 13,309 gene families, of which 433 gene families were unique to *E. angustifolia* (Additional file 1: Table S13). Compared with *Z. jujuba*, *E. angustifolia* had fewer expanded gene families (375 vs. 404) and fewer contracted gene families (464 vs. 469) (Fig. 4A, Additional file 2: Table S14). Enrichment analysis of the 839 expanded and contracted gene families revealed that they were enriched in the pathways strictosidine synthase and xylanase inhibitor N-terminal. Strictosidine synthase has been detected in some major crops and is thought to make crops resistant to salt stress [57]. Xylanase inhibitor N-terminal is thought to be related to thermal stability [58]. The amplification of the above taxonomic genes might be related to the environmental conditions experienced by *E. angustifolia*. High temperature and drought have driven adaptive evolution in *E. angustifolia* [8] and increased evolutionary differences between *E. angustifolia* and other species.

The KEGG results revealed a scattered functional distribution of these genes, and 1 variant gene of the 330 genes was related to the biosynthesis of amino acids (Additional file 1: Table S15). Further analysis of this pathway (Fig. 4B) revealed that the expression of the two pathways was increased during the synthesis of PRPP and imidazole-glycerol-3p. Previous studies indicate that both PRPP and imidazole-glycerol-3p are related to defense [59]. The GO (Additional file 1: Table S15) results showed that 28 variant genes were related to catalytic activity, 36 to metabolic process, and 29 to cellular process. Many variant genes were related to response to stimulus, which might indicate previous natural selection on *E. angustifolia* in response to harsh environments.

Drawing conclusions regarding the reduction in the adaptation of gene families was difficult given that we only analyzed gene families that were partly amplified from *E. angustifolia*. We identified 80 rapidly evolving genes in *E. angustifolia* through comparison of genes with 7 other species (Additional file 3: Table S16). The functional predictions showed that several genes were related to pseudouridine-metabolizing bifunctional protein and RNA pseudouridine synthase. Previous research has shown that pseudouridine-metabolizing bifunctional protein is related to human urinary tract pathogenic *Escherichia coli*, the principal agent of urinary tract infections in humans [60]. Genomic studies of humans and other mammals have shown that there is no gene encoding pseudouridine-metabolizing bifunctional protein, and the ability to metabolize pseudouridine has been lost [60]. This pattern of evolution in *E. angustifolia* might also be related to human and mammalian activities. The gene encoding carbon catabolite repressor protein 4 is also rapidly evolving in *E. angustifolia*. The carbon catabolite repressor 4-CCR4 associated factor1 complex is the major enzyme complex responsible for catalyzing mRNA deadenylation. The degradation of messenger RNA caused by poly (A) tail shortening (deadenylation) is a central mechanism for the biological regulation of gene expression. This is a mechanism that plants have evolved to reprogram gene expression to maintain homeostasis in constantly changing environments [61]. Previous studies investigating carbon catabolite repressor 4-CCR4 associated factor1 function in plants have focused on the role of these genes in mediating biotic stress responses, such as resistance to pathogens [62]. This study has shown that the regulation of the gene encoding carbon catabolite repressor protein 4 has evolved rapidly in *E. angustifolia* to improve its tolerance to abiotic and biotic stress [63]. This is also the first study showing that a gene regulating carbon catabolite repressor protein 4 has been discovered in *E. angustifolia*. Genes related to biotic and abiotic stress resistance were also among some of the 80 rapidly evolving genes, such as peptidyl-prolyl cis–trans isomerase [64].

### Resequencing analysis of 12 wild *E. angustifolia* samples in Gansu Province, China

The resequencing analysis of 12 wild *E. angustifolia* samples in Gansu Province, China (Table 1, Additional file 6: Fig. S1) generated 86.58 Gp clean reads, with a Q20 of 96.64% and Q30 of 91.79%. The average coverage depth was 11 ×, and the genome coverage was 92.89% (Additional file 1: Tables S17–S19). In diploid genomes, runs of homozygosity are uninterrupted homozygous segments in the genome [65], and they can be used to quantify the level of inbreeding either in individuals or populations [66]. In this study, homozygous types accounted for most of the other 11 individuals except for R11 (Table 3). This suggests that hybridization might occur between different close relatives in nature, which is consistent with the observation that hybridization is common in plants [67]. We also detected 74,790 CDS and 1,791,719 genome-wide Indels (Additional file 1: Table S20).

**Table 3:** Number of SNPs for wild *E. angustifolia* samples

Code ID	Sample ID	SNP number	Transition	Transversion	Ti/Tv	Heterozygosity	Homozygosity	Het-ratio (%)
R01	1583	3,097,175	2,110,179	986,996	2.13	1,412,964	1,684,211	45.62
R02	1619	3,223,761	2,197,723	1,026,038	2.14	1,492,655	1,731,106	46.30
R03	1684	3,094,736	2,108,611	986,125	2.13	1,417,978	1,676,758	45.81
R04	1697	3,021,352	2,057,327	964,025	2.13	1,417,892	1,603,460	46.92
R05	1726	3,045,841	2,075,585	970,256	2.13	1,256,764	1,789,077	41.26
R06	1758	3,193,315	2,178,336	1,014,979	2.14	1,442,262	1,751,053	45.16
R07	1784	3,225,579	2,198,583	1,026,996	2.14	1,405,876	1,819,703	43.58
R08	1811	3,108,956	2,117,042	991,914	2.13	1,537,532	1,571,424	49.45
R09	1857	3,022,369	2,058,271	964,098	2.13	1,311,017	1,711,352	43.37
R10	2063	3,028,547	2,060,171	968,376	2.12	1,379,687	1,648,860	45.55
R11	2106	3,223,275	2,197,798	1,025,477	2.14	1,617,799	1,605,476	50.19
R12	2216	3,115,254	2,122,083	993,171	2.13	1,369,366	1,745,888	43.95

In a diploid, if a certain SNP site on homologous chromosomes is the same base, the SNP site is referred to as a homozygous SNP site. If the SNP loci on homologous chromosomes contain different types of bases, the SNP locus is referred to as a heterozygous SNP locus. Greater numbers of homozygous SNPs correspond to greater differences between samples and the reference genome. Greater numbers of heterozygous SNPs correspond to higher degrees of heterozygosity

*number* number of SNPs detected; *Transition* number of SNPs of the conversion type; *Transversion* number of SNPs of transversion type; *Ti/Tv* the ratio of SNPs of conversion to transversion type; *Heterozygosity* number of heterozygous SNPs; *Homozygosity* number of homozygous SNPs; *Het-ratio* the ratio of heterozygous SNPs

**Fig. 5:**
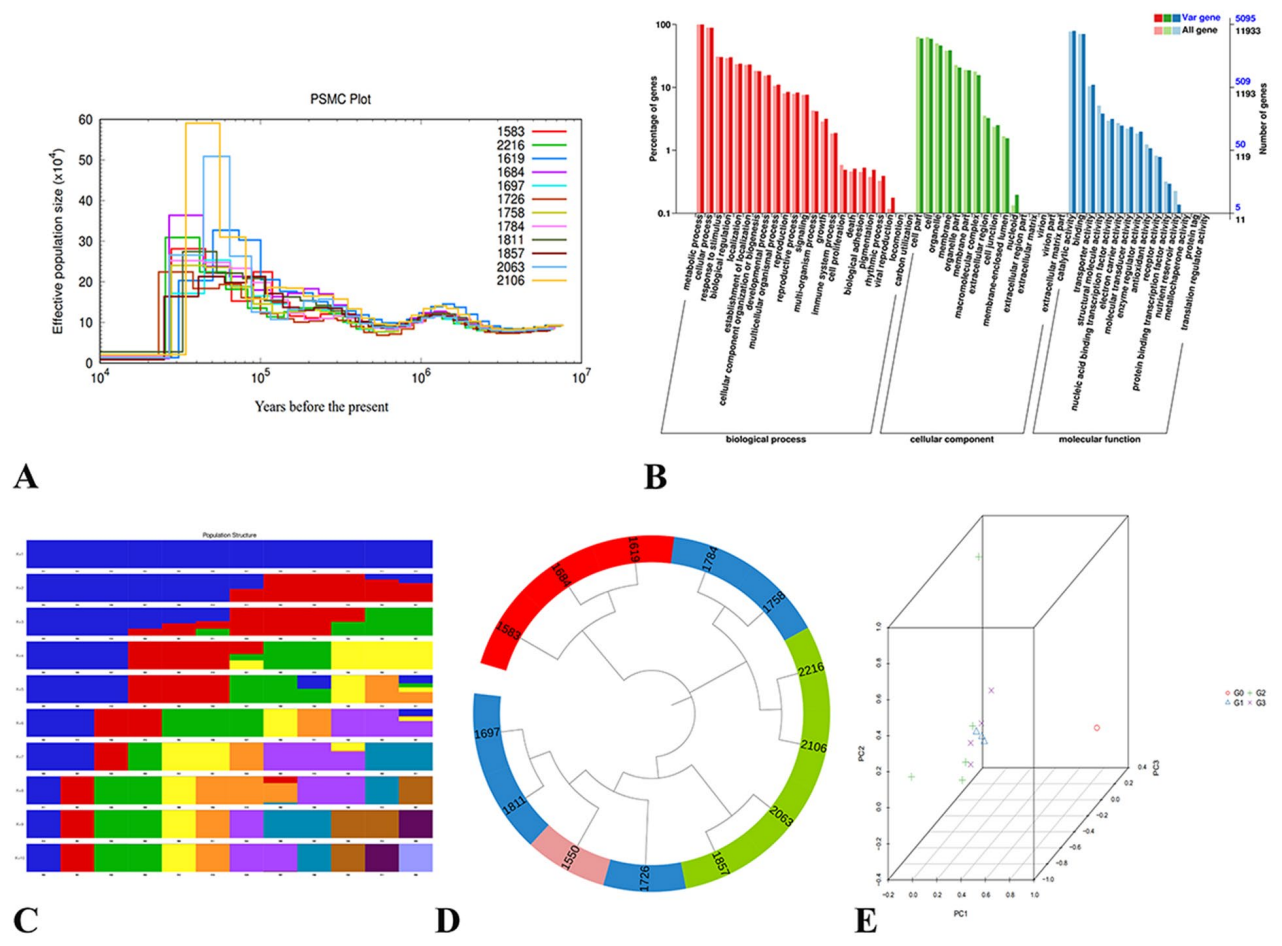
Resequencing analysis. **A** Demographic history of 12 wild *E. angustifolia* samples. **B** GO annotation clustering of variant genes in sample R01. **C** Sample clustering results corresponding to each K value of Admixture. **D** Phylogenetic trees of 12 wild *E. angustifolia* samples and outgroup *Z. jujuba*. The numbers (1583, 1619, 1684, 1697, 1726, 1758, 1784, 1811, 1857, 2063, 2106, 2216) represent the Sample IDs of 12 wild *E. angustifolia* samples, and the number ‘1550’ represents the outgroup *Z. jujuba*. The color of G0, G1, G2, and G3 was purple, red, blue, and green. respectively. **E** Three-way PCA plot of *E. angustifolia* and *Z. jujuba*

The Pairwise Sequentially Markovian Coalescent (PSMC) model was used to estimate the historical effective population size based on genome-wide data of 12 wild *E. angustifolia* species (Fig. 5A). In this study, there was no significant difference in the effective population size among 12 wild *E. angustifolia* species 3.0 Mya, which indicated that there were no evolutionary differences among the 12 *E. angustifolia* 3.0 Mya. This was also similar to the time of differentiation of *A. trichopoda*, the sister of all angiosperms [56]. From 3.0 Mya to 350 thousand years ago (Kya), the effective population size of 12 wild *E. angustifolia* changed slightly. Notably, from 1.5 Mya to 150 Kya, the species occured in Africa and showed no signs of divergence. The effective population size of the genome data of each race in this period was the same [44]. The effective population size of 12 wild *E. angustifolia* changed greatly from 350 to 23 Kya. The largest change was observed in the effective population size of wild *E. angustifolia* R11 (2106), especially in the period 55–35 Kya. The effective population size reached a maximum of approximately 60 × 10^4^. At this time, the global temperature continued to rise, which might promote the rapid growth of the population of *E. angustifolia* [68]. At 23 Kya, the effective population size of 12 wild *E. angustifolia* decreased drastically, which corresponded to the last glacial period (the duration is approximately 26.5 to 19 Kya). The global temperature continued to decrease, and the glaciers at the poles and mountains began to extend to low latitudes and altitudes [69]. This drastic change in the environment might have led to a reduction in the effective population size of *E. angustifolia*.

We used the R01 result to evaluate the function of the mutated genes (Additional file 1: Tables S21, S22, Fig. 5B, C). Substitutions mainly occurred in DNA binding (GO:0003677), Cellular Component: Nucleus (GO:0005634), DNA binding (GO:0003677) related to cytochrome [70], and Cellular Component: Nucleus (GO:0005634) related to defensive T cells [71]. This variation might be related to the environmental conditions experienced by *E. angustifolia*, including water shortages at high altitude [72].

The results of the phylogenetic tree (Fig. 5D) and PCA (Fig. 5E) were not consistent with expectation, with the exception of G1. This might be related to climate change [73] and landform change [74]. Alternatively, differences in the altitude, latitude, and longitude of the sampling sites might explain this pattern (Table 1); various other factors might cause the results to deviate from expectation.

### Genetic mechanisms underlying salt tolerance in *E. angustifolia*

To investigate the genetic mechanisms underlying salt tolerance in *E. angustifolia*, we performed a salinity experiment. We obtained 113.85 Gb clean reads. There were a total of 6.40 Gb clean reads for each sample, and the Q30 base percentage was 90.74% and higher (Additional file 1: Table S23). The clean reads of each sample were aligned to the reference genome, and the alignment efficiency ranged from 90.87 to 92.30% (Additional file 1: Table S24). Based on the comparison results, we carried out an alternative splicing prediction analysis and gene structure optimization analysis. We also compared the genome sequencing results of *E. angustifolia* and identified 4,404 new genes in the transcriptome of *E. angustifolia* (Additional file 4: Table S25), and we obtained 3953 functional annotations (Additional file 1: Table S26). We analyzed the GO pathways functions of the new genes (Additional file 5: Table S27). The results showed that 3391 genes were involved in the regulation of Molecular Function, 3121 genes were involved in the regulation of Biological Process, and 2613 genes were involved in regulation of Cellular Component. In Molecular Function, 256 genes were involved in regulating ATP binding (GO:0005524), 158 genes were involved in regulating metal ion binding (GO:0046872), and 131 genes were involved in regulating zinc ion binding (GO:0008270), 106 genes were involved in regulating the nucleic acid binding (GO:0003676), and 104 genes were involved in regulating the structural constituent of ribosome (GO:0003735). Previous studies have shown that AtABCG36-overexpressing plants are more resistant to drought and salt stress [75], overexpression of metal ion binding peptides–phytochelatins and metallothionein genes increases tolerance to stress [76], and overexpression of GsZFP1 (zinc ion binding) in alfalfa increases salt tolerance [77]. ‘Nucleic acid binding’ was the most significantly enriched GO term in the MF category for both Supreme and Parish up-regulated genes under salt treatment, suggesting that this process might play an important role in salt tolerance in both cultivars [78]; the structural constituent of ribosome has been shown to play an important role in salinity tolerance [79]. In Biological Process, there were 236 genes involved in the regulation of oxidation–reduction process (GO:0055114), 108 genes involved in the regulation of protein phosphorylation (GO:0006468), and 94 genes involved in the regulation of transcription, DNA-templated (GO:0006355). Previous transcriptome profiling experiments have indicated that oxidation–reduction processes, protein phosphorylation, and DNA-templated are related to salt tolerance in plants [80–83]. Xiong et al. [84] suggested that the homeostasis of the oxidation–reduction process is important for salt tolerance in plants. Hsu et al. [85] identified novel salt stress-responsive protein phosphorylation sites from membrane isolates of abiotic-stressed plants by membrane shaving followed by Zr4 + -IMAC enrichment, and the identified phosphorylation sites play an important role in the salt stress response in plants. Wu et al. [78] showed that genes that were down-regulated under salt treatment are involved in “regulation of transcription, DNA-templated.” In Cellular Component, there were 668 genes involved in the regulation of the integral component of membrane (GO:0016021), 201 genes involved in the regulation of nucleus (GO:0005634), 153 genes in the regulation of membrane (GO:0016020), 116 genes involved in regulating cytoplasm (GO:0005737), and 101 genes involved in regulating plasma membrane (GO:0005886). In conclusion, the GO pathways functions of the new genes in *E. angustifolia* showed that the functions of the new genes in the Molecular Function and Biological Process categories were mainly involved in the regulation of salt stress.

**Fig. 6:**
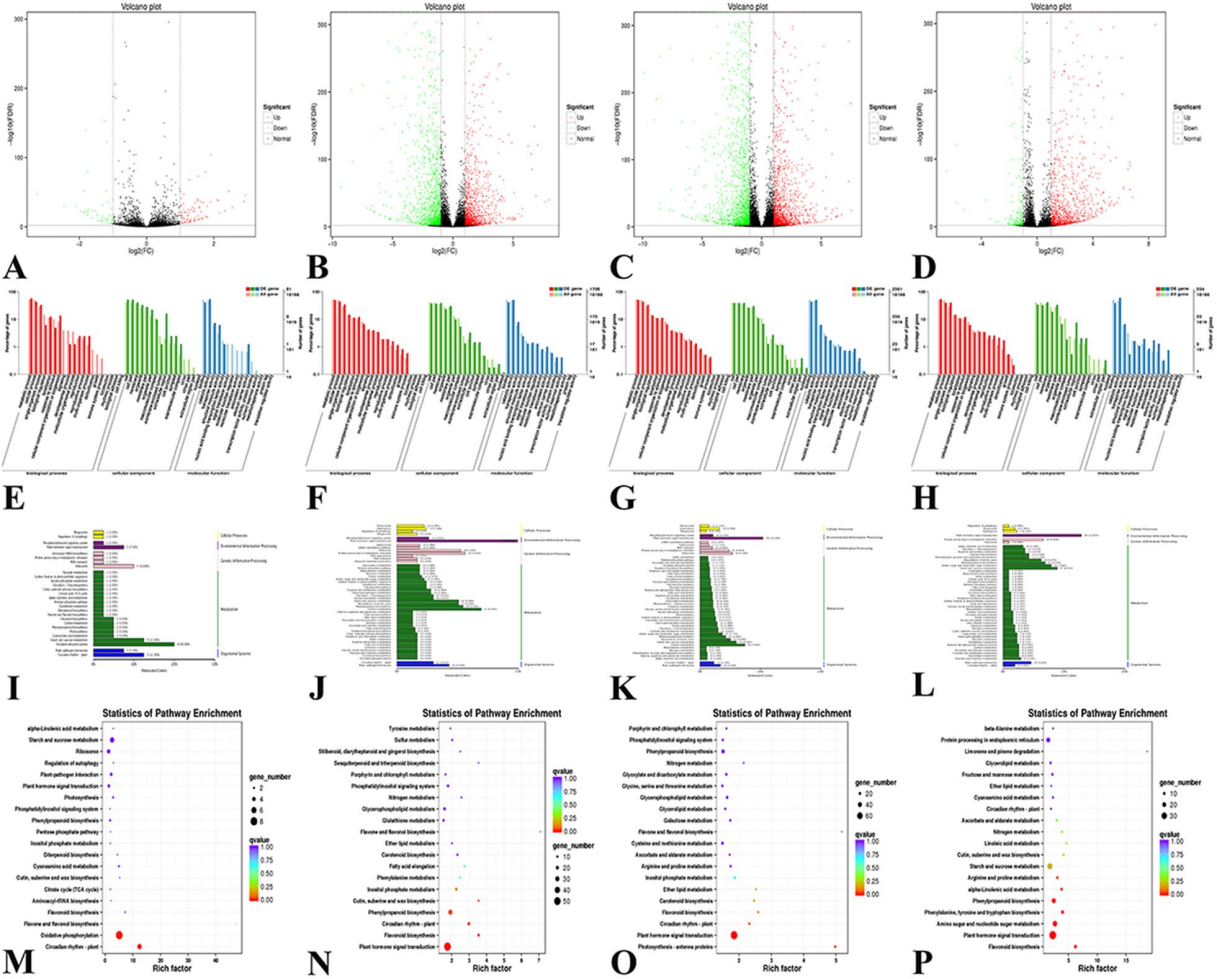
Genetic mechanisms. **A**–**D** Volcano map of differentially expressed transcripts. A, Group 1 vs. Group 2; B, Group 1 vs. Group 3; C, Group 1 vs. Group 4; D, Group 1 vs. Group 5. **E**–**H** GO analysis of the differentially expressed transcripts. E, Group 1 vs. Group 2; F, Group 1 vs. Group 3; G, Group 1 vs. Group 4; H, Group 1 vs. Group 5. **I**–**L** KEGG classification of differently-expressed gene transcripts. I, Group 1 vs. Group 2; J, Group 1 vs. Group 3; K, Group 1 vs. Group 4; L, Group 1 vs. Group 5. **M**–**P** Enrichment and distribution map of the KEGG pathway of differentially expressed genes. M, Group 1 vs. Group 2; N, Group 1 vs. Group 3; O, Group 1 vs. Group 4; P, Group 1 vs. Group 5

According to Additional file 1: Table S28 and Fig. 6A–D, the number of transcripts with significant expression differences between control Group 1 and experimental Group 2 was 137. The expression of 82 and 55 transcripts was significantly up-regulated and down-regulated, respectively. Similarly, the number of transcripts with significant expression differences between control Group 1 and experimental Group 3 was 2670, of which 1260 were up-regulated and 1410 were down-regulated. The number of transcripts with significant expression differences between control Group 1 and experimental Group 4 was 3619, of which 1668 were up-regulated and 1951 were down-regulated. The number of transcripts with significant expression differences between control Group 1 and experimental Group 5 was 1404, of which 1193 were up-regulated and 211 were down-regulated. A large number of differentially expressed transcripts in the two databases indicate that salt stress induces a large number of gene expression changes in *E. angustifolia*, which reflects the complexity of the mechanism underlying the response of *E. angustifolia* to salt stress.

The GO pathway annotations (Fig. 6E–H) of the four experimental groups compared with the control group revealed a pattern consistent with the differential expression among the four experimental groups, and salt stress had a significant effect on proteins in the functional categories Biological Process, Molecular Function, and Cellular Component [86]; the effects on different physiological processes also differed. After salt stress, metabolic process, cellular process, and single-organism process were greatly affected within Biological Process, and binding and catalytic activity were greatly affected within Molecular Function. Salt stress affected the expression of related genes during growth and development as well as the activity of certain proteins [87], which had a greater impact on the growth and development of *E. angustifolia*.

We also performed KEGG pathway analysis of differentially expressed gene transcripts (Fig. 6I–L). The metabolic process and signal pathways that regulate and change under salt stress in plant bodies could be clearly observed according to the KEGG pathway analysis. Comparison of the experimental group and control group revealed that these changes were mostly concentrated in sugar, amino acid metabolism, and plant hormone signal transduction. Salt stress had obvious effects on Environmental Information Process, Genetic Information Processing, and Metabolism and Organismal Systems. After 1 h of salt stress, there were significant changes in oxidative phosphorylation, ribosome, and starch and sucrose metabolism, and KEGG pathways (Fig. 6M) were enriched in oxidative phosphorylation and circadian rhythm-plant. After 6 h and 12 h of salt stress, there were significant changes in plant hormone signal transduction, protein processing in endoplasmic reticulum, biosynthesis of amino acids, phenylpropanoid biosynthesis, carbon metabolism, and plant-pathogen interaction, and KEGG pathways (Fig. 6N, O) were enriched in plant hormone signal transduction. After 24 h of salt stress, there were significant changes in amino sugar and nucleotide sugar metabolism and other metabolic processes, and KEGG pathways (Fig. 6P) were enriched in plant hormone signal transduction.

Several studies have shown that partial KEGG pathway changes are related to salt resistance. Circadian rhythms synchronize intracellular calcium dynamics and ATP production for growth [88] and have a large effect on plant immunity during plant–pathogen interactions [89]. Oxidative phosphorylation plays a role in plant salt tolerance by coordinating ROS scavenging pathways to regulate intracellular ROS levels, prevent cell damage, and control ROS signal transduction [90]. The significance of compatible solutes such as sugars, amino acids, and tertiary amines in salt stress has been well documented [91]. They not only provide an essential source of energy and nutrients for plants under salt stress but also act as osmotic adjustment substances to balance the osmotic potential appended by high salinity [92]. The accumulation of soluble sugars and sucrose can improve salt tolerance [93]. The loss of integrity of the ribosome through the removal of a putative ribosome maturation factor or a ribosomal protein confers salt tolerance to *E. coli* cells [94]. The endoplasmic reticulum is associated with salt tolerance in tomato [95]. Metabolic adaptation is crucial for abiotic stress resistance in plants, and the accumulation of specific amino acids has been suggested to increase tolerance to salt [96]. Enrichment of carbon metabolism and the biosynthesis of amino acids suggests that the synthesis of compatible solutes is fortified to alleviate osmotic stress in plant seedlings under salt treatment [26]. Variation in gene expression under salt stress is also regulated by phytohormones [26]. Changes in the transcription level of hormone genes affect drought and salt stress in plants [97]. de Bruxelles et al. [98] showed that hormones are responsible for changes in the expression of salt-induced genes.

### Conclusions

In this study, the genome of *E. angustifolia* L. was obtained using PacBio technology and Hi-C assisted assembly technology. We estimated the origin of *E. angustifolia* and evaluated its evolutionary relationships with 8 other species. We used comparative genomics to study the adaptive evolution of *E. angustifolia* in Gansu, China. Genes and pathways of salt resistance of *E. angustifolia* were identified through transcriptome analysis. Our findings could be used to aid future comparative genomic analyses of *E. angustifolia* and enhance our understanding of the response of *E. angustifolia* to drought, salt, cold, and wind stress. Our findings also have implications for the planting of *E. angustifolia* and recovery from global land desertification. Several limitations of our study require consideration, given that the findings might be affected by the analytical method used, test conditions, and other environmental conditions; follow-up studies are needed to confirm our findings.

## Supplementary Information

**Additional file 1: Table S1.** Filtering raw data of Pac-bio sequencing. **Table S2.** Length distribution of subreads of Pac-bio sequencing. **Table S3**. Genome assembly evaluation statistics. **Table S4.** Repeating sequence statistics. **Table S5.** Gene information statistics. **Table S6.** Non-coding RNA information. **Table S7.** Gene function annotation statistics. **Table S8.** Statistics of the completeness of the assembled genome. **Table S9.** Statistics of the BUSCO of the assembled genome. **Table S10.** Sequencing data volume statistics. **Table S11.** Clean data and genome alignment results statistics. **Table S12.** Hi-C sequencing data validation. **Table S13.** Classification statistics of gene families. **Table S15.** Function prediction of *E. angustifolia*. **Table S17.** Evaluation statistics of sequencing data of wild *E. angustifolia* samples. **Table S18.** Comparison results of wild *E. angustifolia* samples. **Table S19.** Coverage depth and coverage ratio statistics of wild *E. angustifolia* samples. **Table S20.** InDel statistics of whole genome and coding region of wild *E. angustifolia* samples. **Table S21.** Statistical table of variation genes of wild *E. angustifolia* samples. **Table S22.** Annotated list of some variant genes in sample R01. **Table S23.** Statistics of transcriptome sequencing data. **Table S24.** Results of the comparison between the sequencing transcriptome samples and the genome data. **Table S26.** Statistics of functional annotation results of new genes. **Table S28.** Statistics on the number of different genes in the transcription.

**Additional file 2: Table S14.** Functional gene categories enriched for *E. angustifolia* expansion and contraction families.

**Additional file 3: Table S16.** 80 rapidly evolving genes in *E. angustifolia*.

**Additional file 4: Table S25.** New genes discovered in *E. angustifolia* partially.

**Additional file 5: Table S27.** Go pathways functions of new genes.

**Additional file 6: Figure S1.** 12 wild *E. angustifolia* samples.

## Data Availability

This Whole Genome Shotgun project has been deposited in NCBI. Raw sequencing reads and genome assembly are available at GenBank as BioProject PRJNA647537 (https://dataview.ncbi.nlm.nih.gov/object/PRJNA647537?reviewer=5dse95jg6ae9tao2s0ettacevv). Raw sequencing data (Hi-C, trans data, survey, PB) have been deposited in SRA (Sequence Read Archive) database as SRR12563589–SRR12563593 [99–102]. Resequencing has been deposited in SRA (Sequence Read Archive) database as SRR12578558–SRR12578569 [103]. Transcriptome has been deposited in SRA (Sequence Read Archive) database as SRR12569921–SRR12569935 [104]. Gene annotation data of *E. angustifolia* genome has been deposited in FigShare (https://doi.org/10.6084/m9.figshare.12957110.v1).
