# Supplementary material for: De novo assembly provides new insights into the evolution of Elaeagnus angustifolia L
Source: Plant Methods. 2022 Jun 18;18:84. doi: 10.1186/s13007-022-00915-w (PMC9206267; doi:10.1186/s13007-022-00915-w)
Supplement: Supplementary file 1 — Additional file 1: Table S1. Filtering raw data of Pac-bio sequencing. Table S2. Length distribution of subreads of Pac-bio sequencing. Table S3. Genome assembly evaluation statistics. Table S4. Repeating sequence statistics. Table S5. Gene information statistics. Table S6. Non-coding RNA information. Table S7. Gene function annotation statistics. Table S8. Statistics of the completeness of the assembled genome. Table S9. Statistics of the BUSCO of the assembled genome. Table S10. Sequencing data volume statistics. Table S11. Clean data and genome alignment results statistics. Table S12. Hi-C sequencing data validation. Table S13. Classification statistics of gene families. Table S15. Function prediction of E. angustifolia. Table S17. Evaluation statistics of sequencing data of wild E. angustifolia samples. Table S18. Comparison results of wild E. angustifolia samples. Table S19. Coverage depth and coverage ratio statistics of wild E. angustifolia samples. Table S20. InDel statistics of whole genome and coding region of wild E. angustifolia samples. Table S21. Statistical table of variation genes of wild E. angustifolia samples. Table S22. Annotated list of some variant genes in sample R01. Table S23. Statistics of transcriptome sequencing data. Table S24. Results of the comparison between the sequencing transcriptome samples and the genome data. Table S26. Statistics of functional annotation results of new genes. Table S28. Statistics on the number of different genes in the transcription. [file 13007_2022_915_MOESM1_ESM.docx]

***De novo* assembly provides new insights into the evolution of *Elaeagnus angustifolia* L.**

Yunfei Mao^1^, Xueli Cui^1^, Haiyan Wang^1^, Xin Qin^1^, Yangbo Liu^1^, Yijun Yin^1^, Xiafei Su^1^, Juan Tang^2^, Fengling Wang^2^, Fengwang Ma^3^, Naibin Duan^4^, Donglin Zhang^5^, Yanli Hu^1^, Wenli Wang^1^, Shaochong Wei^1^, Xiaoliu Chen^1^, Zhiquan Mao^1^, Xuesen Chen^1^, Xiang Shen^1,*^.

^1^College of Horticultural Science and Engineering/State Key Laboratory of Crop Biology, Shandong Agricultural University, Tai’an, China.

^2^Biomarker Technologies Corporation, Beijing, China.

^3^College of Horticulture, Northwest Agriculture and Forestry University, Yangling, China.

^4^Germplasm Resource Center of Shandong Province, Shandong Academy of Agricultural Sciences, Jinan, China.

^5^Depart of Horticulture, University of Georgia, Athens, USA.

^*^Corresponding author. Tel +86 13705383303; E-mail shenx@sdau.edu.cn

**Supplementary**

**Table S1**. Filtering raw data of Pac-bio sequencing

| Reads_num | Total_base (bp) | Read_N50 | Mean | Maximum |
| --- | --- | --- | --- | --- |
| 5,125,675 | 44,266,648,415 | 12,635 | 8,636 | 180,400 |

Note: Reads_num: the total number of reads of the sequencing data; Total_base (bp): the total number of bases of the sequencing data; Read N50: the length of the N50 of the sequencing data reads; Mean: the average length of the sequencing data reads; Maximum: the longest reads of the sequencing data length.

**Table S2.** Length distribution of subreads of Pac-bio sequencing

| Length (bp) | Num | Total length (bp) | Average length (bp) |
| --- | --- | --- | --- |
| 500~2000 | 810,173 | 970,639,283 | 1,198.06 |
| 2000~4000 | 786,386 | 2,314,649,723 | 2,943.40 |
| 4000~6000 | 628,209 | 3,121,912,011 | 4,969.54 |
| 6000~8000 | 524,455 | 3,655,919,272 | 6,970.89 |
| 8000~10000 | 472,923 | 4,258,702,118 | 9,005.06 |
| 10000~12000 | 541,115 | 5,950,614,737 | 10,996.95 |
| 12000~14000 | 418,476 | 5,412,976,827 | 12,934.98 |
| 14000~16000 | 283,989 | 4,241,988,099 | 14,937.16 |
| 16000~18000 | 193,862 | 3,283,538,506 | 16,937.50 |
| 18000~ | 466,087 | 11,055,707,839 | 23,720.27 |
| Total | 5,125,675 | 44,266,648,415 | 8,636.26 |

Note: Length (bp): the range of each length of subreads; Num: the number of sequences in each length range; Total length (bp): the total length of the sequence in each length range; Average length (bp): the average length of the sequence.

**Table S3**. Genome assembly evaluation statistics

| Analytical method | Contig number | Contig length (bp) | Contig N50 (bp) | Contig N90 (bp) | Contig max (bp) | GC content (%) | Gap total length (bp) |
| --- | --- | --- | --- | --- | --- | --- | --- |
| Canu | 4,198 | 781,089,350 | 486,923 | 61,763 | 6,883,228 | 30.3 | 0 |
| Falcon | 2,485 | 645,980,163 | 848,862 | 132,200 | 5,717,061 | 30.41 | 0 |
| wtdbg | 903 | 525,523,332 | 4,601,374 | 734,314 | 16,369,342 | 30.35 | 0 |
| Pilon | 523 | 526,802,423 | 12,595,695 | 2,424,952 | 35,818,431 | 30.33 | 0 |

Note: Contig number: the number of Contig above 1 Kb; Contig length (bp): the length of Contig above 1 Kb; Contig N50 (bp): the length of Contig N50 above 1 Kb; Contig N90 (bp): the length of Contig N90 above 1 Kb; Contig max (bp): the length of the longest Contig above 1 Kb; Gap total length (bp): the total length of the gap; GC content (%): GC content.

**Table S4.** Repeating sequence statistics

| Type | Number | Length (bp) | Percentage (%) |
| --- | --- | --- | --- |
| ClassI/DIRS | 57,476 | 39,462,537 | 7.49 |
| ClassI/LINE | 17,420 | 6,130,877 | 1.16 |
| ClassI/LTR | 1,192 | 1,341,892 | 0.25 |
| ClassI/LTR/Copia | 170,211 | 112,045,341 | 21.27 |
| ClassI/LTR/Gypsy | 89,775 | 74,832,142 | 14.2 |
| ClassI/PLE\|LARD | 87,646 | 29,294,594 | 5.56 |
| ClassI/SINE | 3,134 | 580,471 | 0.11 |
| ClassI/TRIM | 4,191 | 2,037,167 | 0.39 |
| ClassI/Unknown | 277 | 111,257 | 0.02 |
| ClassII/Crypton | 10 | 712 | 0 |
| ClassII/Helitron | 9,255 | 2,368,390 | 0.45 |
| ClassII/MITE | 8,168 | 1,544,498 | 0.29 |
| ClassII/Maverick | 1,511 | 278,308 | 0.05 |
| ClassII/TIR | 26,737 | 12,037,464 | 2.29 |
| ClassII/Unknown | 7,008 | 1,957,120 | 0.37 |
| PotentialHostGene | 4,766 | 1,419,371 | 0.27 |
| SSR | 41,290 | 8,338,047 | 1.58 |
| Unknown | 96,908 | 27,036,916 | 5.13 |
| Total without overlap | 626,975 | 263,437,176 | 50.01 |

Note: Type: type of repetitive sequence; Number: number of repetitive sequences obtained; Length (bp): total length of predicted repetitive sequences; Percentage (%): the proportion of repetitive sequences in the total genome.

**Table S5.** Gene information statistics

| Gene Num | GeneLen (bp) | AveGeneLen (bp) | ExonLen (bp) | AveExonLen (bp) | IntronLen (bp) | AveIntronLen (bp) |
| --- | --- | --- | --- | --- | --- | --- |
| 31,730 | 146,984,829 | 4,632.36 | 53,198,808 | 286.56 | 92,673,647 | 602.09 |

Note: Gene Num: predicted number of genes; GeneLen (bp): total length of genes; AveGeneLen (bp): average gene length; ExonLen (bp): total length of exons; AveExonLen (bp): average exon length; IntronLen (bp): total intron length; AveIntronLen (bp): average intron length.

**Table S6.** Non-coding RNA information

| RNA classification | Number | Family |
| --- | --- | --- |
| miRNA | 127 | 24 |
| rRNA | 333 | 4 |
| tRNA | 567 | 24 |

Note: RNA classification: RNA classification; Number: number of predicted RNA; Family: number of RNA family.

**Table S7.** Gene function annotation statistics

| Annotation database | Annotated number | Percentage (%) |
| --- | --- | --- |
| GO | 16,960 | 53.45 |
| KEGG | 11,435 | 36.04 |
| KOG | 17,329 | 54.61 |
| Pfam | 25,462 | 80.25 |
| Swissprot | 22,361 | 70.47 |
| TrEMBL | 30,442 | 95.94 |
| Nr | 30,452 | 95.97 |
| Nt | 30,295 | 95.48 |
| All | 30,743 | 96.89 |

Note: Annotation database: functional annotation database; Annotated number: the number of genes annotated to the corresponding database; Percentage (%): the percentage of genes annotated to the database to the total number of genes.

**Table S8.** Statistics of the completeness of the assembled genome

| Species | Number of 458 CEGs* present in assembly | % of 458 CEGs present in assemblies | Number of 248 highly conserved CEGs present | % of 248 highly conserved CEGs present |
| --- | --- | --- | --- | --- |
| *E. angustifolia* | 446 | 97.38 | 226 | 91.13 |

Note: Species: analyzed species information; Number of 458 CEGs* present in assembly: 428 of the 458 genes in the CEGMA v2.5 database were found in the assembled genome; % of 458 CEGs present in assemblies: included in the assembled genome; Number of 248 highly conserved CEGs present: 237 of the 248 highly conserved genes can be found in the genome; % of 248 highly conserved CEGs present: the proportion of the 237 highly conserved genes in the assembled genome to the total conserved genes.

**Table S9.** Statistics of the BUSCO of the assembled genome

| Species | Complete BUSCOs (C) | Complete and single-copy BUSCOs (S) | Complete and duplicated BUSCOs (D) | Fragmented BUSCOs (F) | Missing BUSCOs (M) | Total Lineage BUSCOs |
| --- | --- | --- | --- | --- | --- | --- |
| *E. angustifolia* | 1,290 | 1,106 | 184 | 23 | 127 | 1440 |

Note: Species: species information analyzed; Complete BUSCOs: number of complete genes found; Complete and single-copy BUSCOs: number of single-copy genes; Complete and duplicated BUSCOs: number of multiple-copy genes; Fragmented BUSCOs: predicted number of incomplete genes; Missing BUSCOs: number of genes that had not been predicted; Total Lineage BUSCOs: number of conserved genes.

**Table S10.** Sequencing data volume statistics

| Read Pairs Number | Base Number | GC Content (%) | %≥Q30 |
| --- | --- | --- | --- |
| 132,177,495 | 39,555,028,064 | 34.68 | 95.46 |

Note: Read Pairs Number: the total number of Pair-end Read in Clean Data; Base Number: the total number of bases in Clean Data; GC Content: the GC content of Clean Data, that is, the G and C bases in Clean Data account for the total bases; %≥Q30: the percentage of bases whose Clean Data quality value is greater than or equal to 30.

**Table S11.** Clean data and genome alignment results statistics

| Mapping Type | Count | Ratio (%) |
| --- | --- | --- |
| Total Read Pairs | 132,177,495 | 100 |
| Mapped Reads | 239,710,117 | 90.68 |
| Unique Mapped Read Pairs | 80,794,872 | 61.13 |

Note: Total Read Pairs: logarithm of clean reads; Mapped Reads: reads aligned to the genome; Unique Mapped Reads Pairs: the only Read Pairs aligned to the genome.

**Table S12.** Hi-C sequencing data validation

| Type | Count | Ratio (%) |
| --- | --- | --- |
| Unique Paired Alignments | 80,794,872 | 100 |
| Valid Interaction Pairs | 72,975,916 | 90.32 |
| Dangling End Pairs | 1,692,503 | 2.09 |
| Re-ligation Pairs | 1,000,553 | 1.24 |
| Self-cycle Pairs | 1,489,390 | 1.84 |
| Dumped Pairs | 3,636,510 | 4.5 |

Note: Unique Paired Alignments: Read Pairs that were uniquely aligned to the genome; Valid Interaction Pairs: Valid Read Pairs; Dangling End Pairs: Read Pairs of the end suspension type in invalid data; Re-ligation Pairs: Adjacent connections in invalid data Type of Read Pairs; Self-circle Ligation Pairs: Invalid data belongs to Read Pairs of self-connection type; Dumped Pairs: Invalid data belongs to other undefined Read Pairs.

**Table S13.** Classification statistics of gene families

| Species name | Total gene number | Cluster gene number | Total family number | Unique gene family number |
| --- | --- | --- | --- | --- |
| *Z.jujuba* | 27,372 | 22,969 | 11,863 | 754 |
| *G.max* | 56,044 | 46,292 | 15,127 | 1,745 |
| *A.thaliana* | 27,369 | 23,168 | 12,738 | 739 |
| *E.angustifolia* | 31,730 | 27,553 | 13,309 | 433 |
| *O.sativa* | 38,852 | 25,569 | 12,465 | 1,989 |
| *P.trichocarpa* | 41,335 | 33,307 | 14,548 | 984 |
| *A.trichopoda* | 16,986 | 15,055 | 11,145 | 247 |
| *S.lycopersicum* | 34,674 | 25,598 | 13,597 | 1,047 |

Note: Species name: species name; Total gene number: the total number of genes; Cluster gene number: the number of genes involved in the clustering of the family; Total family number: how many gene families these genes can be divided into; Unique gene family number: Number of unique gene families.

**Table S15.** Function prediction of *E. angustifolia*

| KEGG Pathway | ko_ID | Variantgene | gene | Variant_gene all | gene_all |  |
| --- | --- | --- | --- | --- | --- | --- |
| Arginine and proline metabolism | ko00330 | 1 | 107 | 18 | 6,482 | EVM0030574.1 |
| Spliceosome | ko03040 | 2 | 287 | 18 | 6,482 | EVM0002033.1; EVM0017987.1 |
| Aminoacyl-tRNA biosynthesis | ko00970 | 1 | 80 | 18 | 6,482 | EVM0023223.1 |
| Glycolysis / Gluconeogenesis | ko00010 | 1 | 170 | 18 | 6,482 | EVM0031123.1 |
| Glycerophospholipid metabolism | ko00564 | 1 | 119 | 18 | 6,482 | EVM0015554.2 |
| RNA degradation | ko03018 | 1 | 175 | 18 | 6,482 | EVM0003593.1 |
| Histidine metabolism | ko00340 | 1 | 30 | 18 | 6,482 | EVM0021419.1 |
| Mismatch repair | ko03430 | 1 | 53 | 18 | 6,482 | EVM0006374.1 |
| Pyruvate metabolism | ko00620 | 1 | 137 | 18 | 6,482 | EVM0023114.1 |
| DNA replication | ko03030 | 1 | 71 | 18 | 6,482 | EVM0006374.1 |
| RNA transport | ko03013 | 1 | 254 | 18 | 6,482 | EVM0006782.1 |
| Biosynthesis of amino acids | ko01230 | 1 | 330 | 18 | 6,482 | EVM0021419.1 |
| Homologous recombination | ko03440 | 2 | 65 | 18 | 6,482 | EVM0000375.1; EVM0006374.1 |
| Terpenoid backbone biosynthesis | ko00900 | 1 | 72 | 18 | 6,482 | EVM0000274.1 |
| Endocytosis | ko04144 | 1 | 217 | 18 | 6,482 | EVM0029705.1 |
| Pyrimidine metabolism | ko00240 | 1 | 155 | 18 | 6,482 | EVM0023652.2 |
| Zeatin biosynthesis | ko00908 | 1 | 27 | 18 | 6,482 | EVM0029302.1 |
| Glycosylphosphatidylinositol (GPI)-anchor biosynthesis | ko00563 | 1 | 28 | 18 | 6,482 | EVM0021465.1 |
| Base excision repair | ko03410 | 1 | 54 | 18 | 6,482 | EVM0006650.1 |

| GO pathway | gene_all | Variant_gene all |
| --- | --- | --- |
| cellular component |  |  |
| extracellular region | 396 | 0 |
| cell | 7,054 | 23 |
| nucleoid | 15 | 1 |
| membrane | 4,277 | 5 |
| virion | 6 | 0 |
| cell junction | 260 | 0 |
| extracellular matrix | 6 | 0 |
| membrane-enclosed lumen | 188 | 0 |
| macromolecular complex | 2,015 | 2 |
| organelle | 5,608 | 21 |
| extracellular matrix part | 1 | 0 |
| extracellular region part | 7 | 0 |
| organelle part | 2,548 | 5 |
| virion part | 6 | 0 |
| membrane part | 2,118 | 2 |
| cell part | 7,097 | 23 |
| molecular function |  |  |
| protein binding transcription factor activity | 35 | 0 |
| nucleic acid binding transcription factor activity | 333 | 0 |
| catalytic activity | 8,619 | 28 |
| receptor activity | 92 | 0 |
| structural molecule activity | 575 | 0 |
| transporter activity | 1,174 | 2 |
| binding | 7,895 | 18 |
| electron carrier activity | 310 | 0 |
| antioxidant activity | 141 | 0 |
| metallochaperone activity | 4 | 0 |
| enzyme regulator activity | 205 | 0 |
| protein tag | 2 | 0 |
| translation regulator activity | 1 | 0 |
| nutrient reservoir activity | 26 | 0 |
| molecular transducer activity | 246 | 0 |
| biological process |  |  |
| reproduction | 894 | 3 |
| immune system process | 200 | 0 |
| metabolic process | 1,1165 | 36 |
| cell proliferation | 67 | 0 |
| cellular process | 9,948 | 29 |
| carbon utilization | 7 | 0 |
| viral reproduction | 13 | 0 |
| death | 51 | 0 |
| reproductive process | 876 | 3 |
| biological adhesion | 49 | 0 |
| signaling | 841 | 1 |
| multicellular organismal process | 1,175 | 3 |
| developmental process | 1,715 | 7 |
| growth | 312 | 0 |
| locomotion | 7 | 0 |
| pigmentation | 44 | 0 |
| rhythmic process | 38 | 0 |
| response to stimulus | 3,431 | 8 |
| localization | 2,623 | 3 |
| establishment of localization | 2,549 | 3 |
| multi-organism process | 470 | 1 |
| biological regulation | 3,266 | 8 |
| cellular component organization or biogenesis | 2,065 | 4 |

**Table S17.** Evaluation statistics of sequencing data of wild *E. angustifolia* samples

| Code ID | Sample ID | Raw_Reads | Clean_Reads | Clean_Base | Q20 (%) | Q30 (%) | GC (%) |
| --- | --- | --- | --- | --- | --- | --- | --- |
| R01 | 1583 | 23,121,524 | 23,117,711 | 6,927,008,434 | 97.89 | 93.75 | 30.96 |
| R02 | 1619 | 31,013,143 | 31,010,590 | 9,292,470,648 | 97.09 | 91.87 | 31.02 |
| R03 | 1684 | 23,608,952 | 23,606,400 | 7,073,703,948 | 96.9 | 91.41 | 31.04 |
| R04 | 1697 | 21,700,692 | 21,699,427 | 6,502,099,942 | 97.03 | 91.77 | 31.59 |
| R05 | 1726 | 23,039,258 | 23,037,842 | 6,903,361,770 | 96.75 | 91.17 | 31.72 |
| R06 | 1758 | 22,464,409 | 22,462,745 | 6,730,874,032 | 97.1 | 91.88 | 31.14 |
| R07 | 1784 | 25,326,698 | 25,324,149 | 7,588,447,532 | 97.02 | 91.72 | 31.19 |
| R08 | 1811 | 24,836,374 | 24,834,931 | 7,441,757,850 | 96.91 | 91.4 | 31.11 |
| R09 | 1857 | 22,188,727 | 22,185,119 | 6,647,816,544 | 97.36 | 92.35 | 31.21 |
| R10 | 2063 | 24,277,698 | 24,276,211 | 7,274,311,306 | 96.96 | 91.55 | 31.22 |
| R11 | 2106 | 26,032,662 | 26,030,738 | 7,800,221,826 | 96.97 | 91.54 | 30.84 |
| R12 | 2216 | 21,353,102 | 21,351,702 | 6,398,196,172 | 96.74 | 91.11 | 31.19 |

Note: Raw_Reads: the number of initial sequencing reads, with four rows in a unit to count the number of pair-end sequences; Clean_Reads: the number of filtered reads, the calculation method is the same as Raw Reads; Clean_base: the number of filtered bases, the number of Clean Reads is multiplied by Sequence length; Q20 (%): the percentage of bases with a quality value greater than or equal to 20 to the total number of bases; Q30 (%): the percentage of bases with a quality value greater than or equal to 30 to the total number of bases; GC (%): Sample GC content, that is, the percentage of bases of type G and C to the total bases.

**Table S18.** Comparison results of wild *E. angustifolia* samples

| Code ID | Sample ID | Total_reads | Mapped (%) | Properly_mapped (%) |
| --- | --- | --- | --- | --- |
| R01 | 1583 | 46,235,422 | 96.54 | 93.29 |
| R02 | 1619 | 62,021,180 | 96.66 | 92.72 |
| R03 | 1684 | 47,212,800 | 96.79 | 92.93 |
| R04 | 1697 | 43,398,854 | 95.83 | 92.19 |
| R05 | 1726 | 46,075,684 | 97.04 | 92.87 |
| R06 | 1758 | 44,925,490 | 96.82 | 93.29 |
| R07 | 1784 | 50,648,298 | 96.67 | 92.69 |
| R08 | 1811 | 49,669,862 | 96.64 | 93.12 |
| R09 | 1857 | 44,370,238 | 96.75 | 93.12 |
| R10 | 2063 | 48,552,422 | 97.03 | 93.25 |
| R11 | 2106 | 52,061,476 | 96.77 | 93.3 |
| R12 | 2216 | 42,703,404 | 96.14 | 92.24 |

Note: Total_Reads: the number of Clean Reads; Mapped (%): the percentage of the number of Clean Reads mapped to the reference genome to the number of all Clean Reads; Properly mapped: paired-end sequencing sequences are all mapped to the reference genome and the distance is consistent with the length distribution of the sequenced fragments.

**Table S19.** Coverage depth and coverage ratio statistics of wild *E. angustifolia* samples

| Code ID | Sample ID | Ave_depth | Cov_ratio_1X (%) | Cov_ratio_5X (%) | Cov_ratio_10X (%) |
| --- | --- | --- | --- | --- | --- |
| R01 | 1583 | 12 | 92.78 | 85.51 | 61.8 |
| R02 | 1619 | 15 | 93.32 | 88.47 | 76.49 |
| R03 | 1684 | 12 | 92.9 | 85.45 | 61.43 |
| R04 | 1697 | 10 | 92.8 | 83.18 | 53.39 |
| R05 | 1726 | 11 | 92.52 | 83.27 | 55.96 |
| R06 | 1758 | 11 | 92.68 | 83.99 | 58.03 |
| R07 | 1784 | 12 | 92.98 | 86.08 | 64.83 |
| R08 | 1811 | 12 | 93.21 | 86.44 | 65.36 |
| R09 | 1857 | 11 | 92.73 | 84.62 | 57.42 |
| R10 | 2063 | 11 | 92.83 | 85.22 | 60.3 |
| R11 | 2106 | 13 | 93.28 | 87.35 | 69.92 |
| R12 | 2216 | 10 | 92.65 | 83.04 | 52.61 |

Note: Ave-depth: the average coverage depth of the sample; Cov_ratio_*: the ratio of the number of bases whose coverage depth is at a given depth and above to the total bases of the reference genome.

**Table S20.** InDel statistics of whole genome and coding region of wild *E. angustifolia* samples

| Code ID | Sample ID | CDS Insertion | CDS Deletion | CDS Homo | CDS Het | CDS Total | Genome Insertion | Genome Deletion | Genome Homo | Genome Het | Genome Total |
| --- | --- | --- | --- | --- | --- | --- | --- | --- | --- | --- | --- |
| R01 | 1,583 | 23,825 | 19,193 | 23,502 | 19,516 | 43,018 | 543,351 | 460,382 | 528,887 | 474,846 | 1,003,733 |
| R02 | 1,619 | 24,596 | 19,910 | 23,701 | 20,805 | 44,506 | 565,971 | 478,470 | 538,850 | 505,591 | 1,044,441 |
| R03 | 1,684 | 23,640 | 18,998 | 22,678 | 19,960 | 42,638 | 540,337 | 452,952 | 533,162 | 460,127 | 993,289 |
| R04 | 1,697 | 22,739 | 18,583 | 22,120 | 19,202 | 41,322 | 513,866 | 434,466 | 505,528 | 442,804 | 948,332 |
| R05 | 1,726 | 22,511 | 18,054 | 23,913 | 16,652 | 40,565 | 520,489 | 437,749 | 549,620 | 408,618 | 958,238 |
| R06 | 1,758 | 23,437 | 18,833 | 23,810 | 18,460 | 42,270 | 540,865 | 460,632 | 537,456 | 464,041 | 1,001,497 |
| R07 | 1,784 | 24,516 | 19,747 | 23,361 | 20,902 | 44,263 | 559,815 | 476,300 | 550,599 | 485,516 | 1,036,115 |
| R08 | 1,811 | 23,773 | 19,225 | 21,729 | 21,269 | 42,998 | 539,896 | 456,646 | 491,178 | 505,364 | 996,542 |
| R09 | 1,857 | 23,324 | 18,854 | 23,288 | 18,890 | 42,178 | 528,249 | 443,545 | 545,143 | 426,651 | 971,794 |
| R10 | 2,063 | 23,734 | 19,114 | 22,498 | 20,350 | 42,848 | 531,899 | 447,552 | 520,451 | 459,000 | 979,451 |
| R11 | 2,106 | 24,276 | 19,673 | 22,472 | 21,477 | 43,949 | 557,357 | 472,856 | 504,721 | 525,492 | 1,030,213 |
| R12 | 2,216 | 23,498 | 19,010 | 23,913 | 18,595 | 42,508 | 534,295 | 451,263 | 548,960 | 436,598 | 985,558 |
| Total | 37,567 | 37,223 | -- | -- | 74,790 | 879,158 | 912,561 | -- | -- | 1,791,719 |  |

Note: CDS: InDel statistics of coding region; Genome: genome-wide InDel statistics; Insertion: number of detected insertions; Delete: number of detected deletions; Het: number of heterozygous InDel; Homo: number of homozygous InDel; Total: Total number of detected InDel (duplicate removal).

**Table S21.** Statistical table of variation genes of wild *E. angustifolia* samples

| Code ID | Sample ID | Gene Number |
| --- | --- | --- |
| R01 | 1583 | 13,104 |
| R02 | 1619 | 13,256 |
| R03 | 1684 | 22,001 |
| R04 | 1697 | 12,911 |
| R05 | 1726 | 12,827 |
| R06 | 1758 | 13,205 |
| R07 | 1784 | 17,581 |
| R08 | 1811 | 13,136 |
| R09 | 1857 | 13,143 |
| R10 | 2063 | 12,974 |
| R11 | 2106 | 13,181 |
| R12 | 2216 | 13,067 |

**Table S22.** Annotated list of some variant genes in sample R01

| Gene ID | GO_annotation | KEGG_annotation | nr_annotation | nt_annotation |
| --- | --- | --- | --- | --- |
| EVM0000007.1 | -- | -- | PREDICTED: nuclear export mediator factor Nemf isoform X1 [Prunus mume] | *Morus notabilis* Nuclear export mediator factor Nemf partial mRNA |
| EVM0000014.1 | Molecular Function: DNA binding (GO:0003677); Cellular Component: nucleus (GO:0005634); | -- | PREDICTED: MADS-box transcription factor 25 isoform X1 [Pyrus x bretschneideri] | PREDICTED: *Malus* x *domestica* MADS-box transcription factor 25-like (LOC103451806), transcript variant X1, mRNA |
| EVM0000015.1 | Biological Process: anion transport (GO:0006820); | -- | Guard cell S-type anion channel SLAC1 [Morus notabilis] | PREDICTED: *Vitis vinifera* guard cell S-type anion channel SLAC1 (LOC100244459), mRNA |
| EVM0000016.1 | -- | -- | hypothetical protein PRUPE_ppa002460mg [Prunus persica] | *Prunus persica* hypothetical protein (PRUPE_ppa002460mg) mRNA, complete cds |

**Table S23.** Statistics of transcriptome sequencing data

| Samples | Clean reads | Clean bases | GC Content | %≥Q30 |
| --- | --- | --- | --- | --- |
| T01 | 25,290,427 | 7,555,724,612 | 43.66% | 91.46% |
| T02 | 22,971,056 | 6,862,736,894 | 43.47% | 91.73% |
| T03 | 27,567,183 | 8,230,149,772 | 43.36% | 91.91% |
| T04 | 28,580,320 | 8,540,911,272 | 43.34% | 92.05% |
| T05 | 30,241,078 | 9,021,152,766 | 43.59% | 92.20% |
| T06 | 26,423,128 | 7,886,658,548 | 43.29% | 92.04% |
| T07 | 27,864,098 | 8,323,839,274 | 43.15% | 91.97% |
| T08 | 21,809,833 | 6,489,004,152 | 42.72% | 91.35% |
| T09 | 23,788,109 | 7,107,686,066 | 43.03% | 91.09% |
| T10 | 25,504,717 | 7,616,338,952 | 43.21% | 91.31% |
| T11 | 26,123,821 | 7,799,874,588 | 43.18% | 91.33% |
| T12 | 22,371,294 | 6,684,747,652 | 43.26% | 91.68% |
| T13 | 29,659,183 | 8,843,785,834 | 43.38% | 91.67% |
| T14 | 21,719,917 | 6,489,395,790 | 43.32% | 90.74% |
| T15 | 21,430,083 | 6,400,801,888 | 43.46% | 91.84% |

**Table S24.** Results of the comparison between the sequencing transcriptome samples and the genome data

| Samples | Total Reads | Mapped Reads | Uniq Mapped Reads | Multiple Map Reads | Reads Map to '+' | Reads Map to '-' |
| --- | --- | --- | --- | --- | --- | --- |
| T01 | 50,580,854 | 46,150,144 (91.24%) | 42,174,894 (83.38%) | 3,975,250 (7.86%) | 22,147,420 (43.79%) | 22,316,140 (44.12%) |
| T02 | 45,942,112 | 41,958,032 (91.33%) | 38,565,059 (83.94%) | 3,392,973 (7.39%) | 20,265,237 (44.11%) | 20,397,800 (44.40%) |
| T03 | 55,134,366 | 50,368,930 (91.36%) | 46,481,745 (84.31%) | 3,887,185 (7.05%) | 24,449,891 (44.35%) | 24,560,158 (44.55%) |
| T04 | 57,160,640 | 52,606,400 (92.03%) | 48,954,601 (85.64%) | 3,651,799 (6.39%) | 25,806,375 (45.15%) | 25,836,248 (45.20%) |
| T05 | 60,482,156 | 55,826,534 (92.30%) | 51,557,716 (85.24%) | 4,268,818 (7.06%) | 27,126,285 (44.85%) | 27,236,767 (45.03%) |
| T06 | 52,846,256 | 48,659,945 (92.08%) | 45,327,432 (85.77%) | 3,332,513 (6.31%) | 23,889,623 (45.21%) | 23,916,678 (45.26%) |
| T07 | 55,728,196 | 51,305,618 (92.06%) | 47,311,479 (84.90%) | 3,994,139 (7.17%) | 24,863,735 (44.62%) | 25,028,377 (44.91%) |
| T08 | 43,619,666 | 39,825,510 (91.30%) | 36,841,092 (84.46%) | 2,984,418 (6.84%) | 19,371,641 (44.41%) | 19,489,286 (44.68%) |
| T09 | 47,576,218 | 43,535,764 (91.51%) | 40,069,749 (84.22%) | 3,466,015 (7.29%) | 21,049,420 (44.24%) | 21,217,959 (44.60%) |
| T10 | 51,009,434 | 46,431,553 (91.03%) | 42,769,144 (83.85%) | 3,662,409 (7.18%) | 22,439,711 (43.99%) | 22,642,918 (44.39%) |
| T11 | 52,247,642 | 47,476,336 (90.87%) | 43,783,600 (83.80%) | 3,692,736 (7.07%) | 23,031,562 (44.08%) | 23,206,010 (44.42%) |
| T12 | 44,742,588 | 40,680,840 (90.92%) | 37,387,699 (83.56%) | 3,293,141 (7.36%) | 19,612,599 (43.83%) | 19,801,298 (44.26%) |
| T13 | 59,318,366 | 54,484,349 (91.85%) | 49,922,781 (84.16%) | 4,561,568 (7.69%) | 26,188,679 (44.15%) | 26,456,147 (44.60%) |
| T14 | 43,439,834 | 39,725,626 (91.45%) | 36,504,242 (84.03%) | 3,221,384 (7.42%) | 19,203,627 (44.21%) | 19,347,077 (44.54%) |
| T15 | 42,860,166 | 39,425,661 (91.99%) | 35,995,761 (83.98%) | 3,429,900 (8.00%) | 18,898,254 (44.09%) | 19,101,331 (44.57%) |

Note: Total Reads: Clean Reads number, according to single end; Mapped Reads: the number of Reads compared to the reference genome and the percentage of Reads in Clean Reads; Uniq Mapped Reads: the number of Reads Mapped to the unique position of the reference genome and the percentage of Reads in Clean Reads; Multiple Map Reads: the number of Reads to Multiple locations of the reference genome and the percentage of Reads in Clean Reads were compared; Reads Map to '+': the number of Reads to the reference genome plus strand and the percentage of Reads in Clean Reads were compared; Reads Map to '-': the number of Reads to the negative strand of the reference genome and the percentage of Reads in Clean Reads.

**Table S26.** Statistics of functional annotation results of new genes

| Annotated databases | New Gene Number |
| --- | --- |
| COG | 1,233 |
| GO | 2,619 |
| KEGG | 1,575 |
| KOG | 2,219 |
| Pfam | 2,867 |
| Swiss-Prot | 2,952 |
| eggNOG | 3,789 |
| nr | 3,943 |

**Table S28.** Statistics on the number of different genes in the transcription

| group | DEGs_total | DEGs_up | DEGs_down |
| --- | --- | --- | --- |
| Group 1 vs Group 2 | 137 | 82 | 55 |
| Group 1 vs Group 3 | 2,670 | 1,260 | 1,410 |
| Group 1 vs Group 4 | 3,619 | 1,668 | 1,951 |
| Group 1 vs Group 5 | 1,404 | 1,193 | 211 |
