# Supplementary material for: De novo assembly provides new insights into the evolution of Elaeagnus angustifolia L
Source: Plant Methods. 2022 Jun 18;18:84. doi: 10.1186/s13007-022-00915-w (PMC9206267; doi:10.1186/s13007-022-00915-w)
Supplement: Supplementary file 6 — Additional file 6: Figure S1. 12 wild E. angustifolia samples. [file 13007_2022_915_MOESM6_ESM.doc]

***De novo* assembly provides new insights into the evolution of *Elaeagnus angustifolia* L.**

Yunfei Mao^1^, Xueli Cui^1^, Haiyan Wang^1^, Xin Qin^1^, Yangbo Liu^1^, Yijun Yin^1^, Xiafei Su^1^, Juan Tang^2^, Fengling Wang^2^, Fengwang Ma^3^, Naibin Duan^4^, Donglin Zhang^5^, Yanli Hu^1^, Wenli Wang^1^, Shaochong Wei^1^, Xiaoliu Chen^1^, Zhiquan Mao^1^, Xuesen Chen^1^, Xiang Shen^1,*^.

^1^College of Horticultural Science and Engineering/State Key Laboratory of Crop Biology, Shandong Agricultural University, Tai’an, China.

^2^Biomarker Technologies Corporation, Beijing, China.

^3^College of Horticulture, Northwest Agriculture and Forestry University, Yangling, China.

^4^Germplasm Resource Center of Shandong Province, Shandong Academy of Agricultural Sciences, Jinan, China.

^5^Depart of Horticulture, University of Georgia, Athens, USA.

^*^Corresponding author. Tel +86 13705383303; E-mail shenx@sdau.edu.cn

**Supplementary**

**
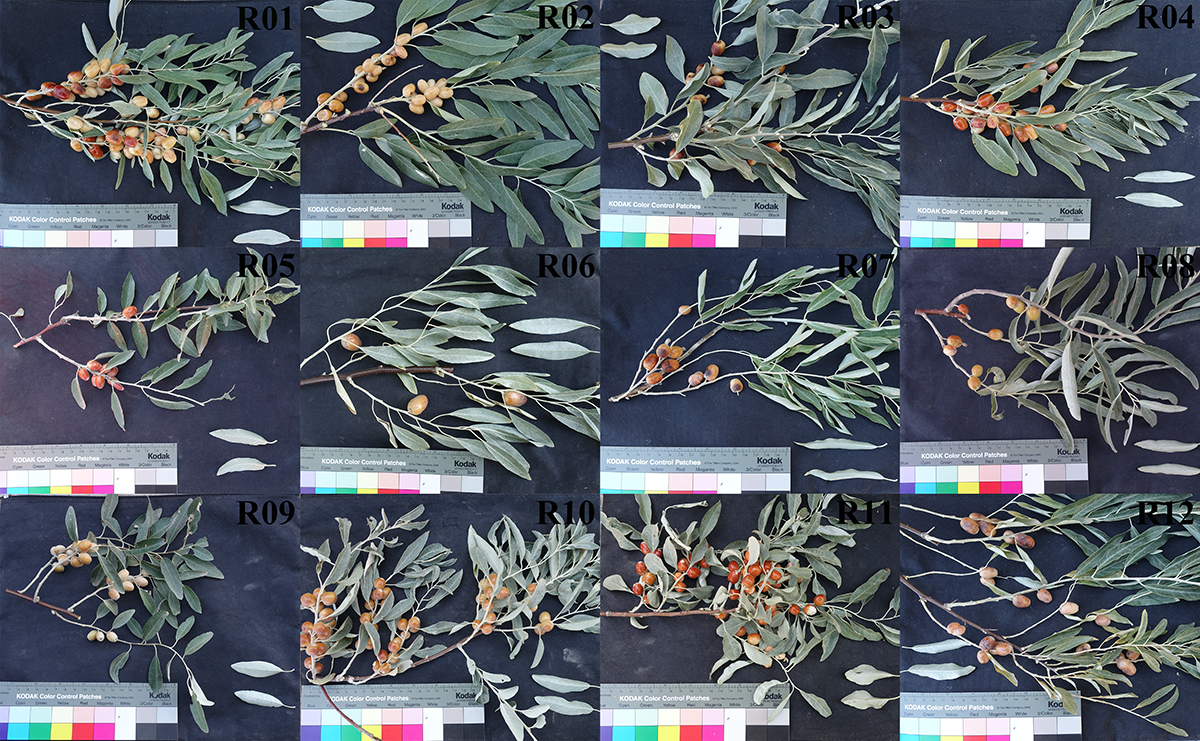
**

**Fig. S1** 12 wild *E. angustifolia* samples. The leaves and fruits of each sample.
